# Quantification of Antiretroviral Drug Emtricitabine in Human Plasma by Surface Enhanced Raman Spectroscopy

**DOI:** 10.1021/acsomega.4c06162

**Published:** 2024-11-25

**Authors:** Marguerite R. Butler, Terry A. Jacot, Sucharita M. Dutta, Gustavo F. Doncel, John B. Cooper

**Affiliations:** † Department of Chemistry and Biochemistry, Old Dominion University, Norfolk, Virginia 23529, United States; ‡ CONRAD, Eastern Virginia Medical School, Norfolk, Virginia 23507, United States

## Abstract

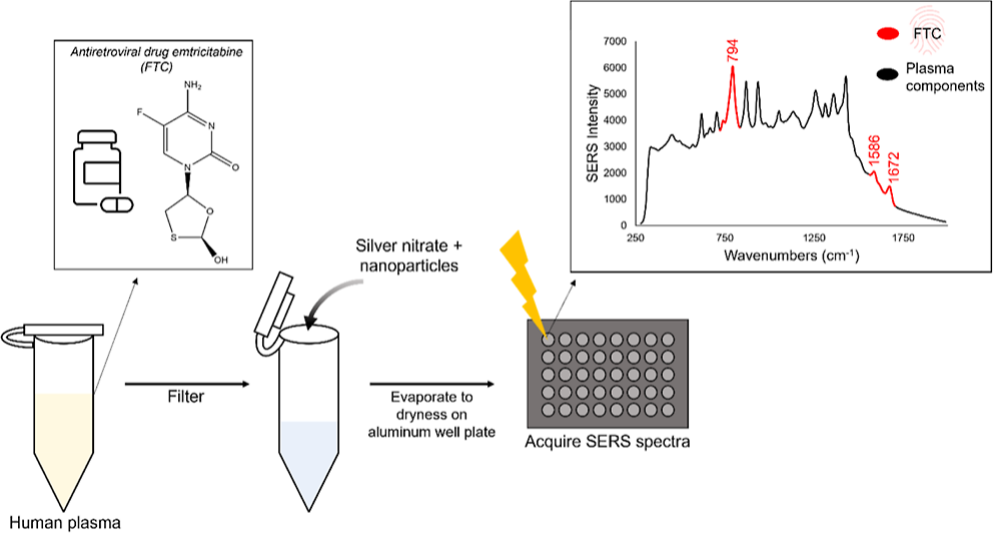

In this study, reproducible label-free detection and quantification of the antiretroviral drug emtricitabine (FTC) down to 78 ng/mL in human plasma by surface enhanced Raman spectroscopy (SERS) is presented. A novel plasma sample pretreatment method using silver nitrate and silver colloidal nanoparticles (Ag CNPs) was used to prepare the plasma samples for analysis. The pretreated plasma samples were evaporated to dryness on an aluminum surface and a computer-controlled Raman scanning system was used to collect spatially resolved SERS spectra of the entire surface. Calibration curves of commercial human plasma samples containing FTC in a concentration range of 5000 to 78 ng/mL were calculated using three different methods. First, a conventional approach was taken, where all the spectra collected for each concentration were averaged, then the SERS intensity of a known FTC peak (792 cm^–1^) was used for calibrations (total population method). This approach was refined by utilizing a figure-of-merit (FOM) quality index (*Q*_i_) to sample spectra from each concentration that contained the highest signal-to-noise (S/N), before averaging and calculating the SERS intensity of the 792 cm^–1^ FTC peak (*Q*_i_ sample method). Finally, the distribution of all *Q*_i_ values for each concentration were modeled using cumulative distribution functions (CDFs) and were used for calibrations (CDF method). The CDF method exhibited the highest analytical sensitivity (slope = 3702.47) compared to the *Q*_i_ sample method (slope = 1591.05) and the total population method (slope = 754.21). The *Q*_i_ sample method exhibited the highest linearity (*R*^2^ = 0.99) compared to the CDF method (*R*^2^ = 0.95) and the total population average (*R*^2^ = 0.97). The CDF method exhibited the highest S/N in the concentration range of 5000 to 312 ng/mL (S/N range of 31.5–16.6). The *Q*_i_ sample method exhibited the highest S/N for concentrations 156 and 78 ng/mL (S/N = 9.7 and 7.4, respectively). These results show that the *Q*_i_ sample method is advantageous over all other methods when approaching the LOQ while the CDF method is advantageous over all methods at higher concentrations. The LOQ (78 ng/mL) was confirmed by principal component analysis (PCA). Together these results show that statistical treatment of a large population of SERS spectra, where the analyte signal intensity follows an exponential distribution, is superior to standard methods of averaging populations of spectra in terms of analytical sensitivity, linearity, and S/N. Additionally, it was found that the background signal had no interference with the quantitative data calculated for the total population and *Q*_i_ sample methods after repeating both analyses with baseline-subtracted spectra. The results and methodology presented in this study establish a framework for integrating SERS into drug adherence monitoring for FTC-based treatment and prevention of infections by demonstrating consistent SERS detection and quantification of FTC in human plasma at therapeutically relevant concentrations.

## 1 Introduction

Medication adherence, also referred to as medication compliance, can be defined as the degree to which a patient follows their prescribed medication regimen in terms of timing, dosage, and frequency.^1^ Proper medication adherence is particularly critical to maintain the efficacy of human immunodeficiency virus (HIV) antiretroviral therapy (ART). Failure to adhere to ART medication regimens can result in decreased viral suppression, emergence of ART-resistant strains, increased therapy cost, and decreased therapeutic options.^2^ Additionally, medication regimens for HIV pre-exposure prophylaxis (PrEP) have been approved by the US Food and Drug Administration (FDA) to reduce new HIV infections among at-risk patients.^3^ There has been suboptimal medication adherence reported for PrEP across different socioeconomic populations, reducing the preventative benefits these regimens provide.^3^ Laboratory tests for assessing the level of adherence to a PrEP or ART regimen typically consist of quantifying drug metabolite accumulation levels in different specimens, including urine, blood, and hair.^4,5^ Complex instrumentation, including liquid chromatography with tandem mass spectrometry (LC–MS/MS), is used for this analysis.^4^ Because of the high cost and level of expertise this instrumentation requires for effective analysis, several groups have made efforts toward developing alternative point of care assays to assess HIV ART and PrEP drug regimen adherence, including lateral flow immunoassays.^6^ Surface enhanced Raman spectroscopy (SERS) has remained a relatively unexplored method as a portable and rapid means of assessing PrEP and ART drug regimen adherence, presumably due to limitations listed above.

SERS has been growing rapidly in popularity as a potential tool for clinical applications. SERS is akin to Raman spectroscopy, which is a technique that involves the detection of scattered photons whose energies are different from the incident photons (known as the Raman Effect).^7^ Particularly, this process is caused by inelastic scattering of photons, where these changes in photon energy are from interactions with vibrating molecules. As a result of these interactions, the resulting Raman spectrum for a given molecule is unique (oftentimes called a “fingerprint” spectrum), allowing one to extract valuable vibrational and structural information. Unlike the complementary technique infrared (IR) spectroscopy, Raman spectroscopy is capable of measuring aqueous samples without detrimental water interference, making it highly suitable for biological applications. However, a calamitous downside of Raman spectroscopy is its inherent lack of sensitivity where for every 10^7^ elastically scattered photons there is only one inelastically scattered photon encoded with molecular data.^8^ SERS utilizes electromagnetic field enhancements provided by small metallic nanostructures to enhance Raman scattering, achieving enhancement factors as high as 10^10^.^9^

In recent years, there has been an increase of studies on developing and utilizing SERS platforms as a point of care diagnostic tool. For example, hand-held Raman devices have been developed and used in point of care settings to diagnose different respiratory viruses.^10,11^ Wearable noninvasive SERS sensors for monitoring different conditions, including wound healing stages^12^ and glucose levels,^13^ have also been reported. Additionally, SERS substrates themselves are increasingly demonstrating their versatility in biomedical applications. Recent developments include improved control of photothermal therapy with real-time temperature monitoring using lanthanide-gold nanoparticles,^14^ dual-mode biosensors combining SERS and field effect transistor (FET) technologies for the detection of toxins in drinking water,^15^ and SERS membranes for the ultrasensitive detection of environmental carcinogenic agents.^16^

Being one of the most widely collected specimens, there is a multitude of SERS studies on blood and other blood products (i.e., serum and plasma) in literature, ranging from forensic^17^ to early cancer diagnosis^18^ applications. While studies of this nature have exceptional specificity for the target biomolecule thanks to labeling techniques (i.e., using Raman reporters, aptamers, or antibodies), high cost and expertise requirements for procurement or synthesis of these materials are drawbacks toward widespread implementation.^19^ Alternatively, SERS-based clinical diagnostics can be achieved in a label-free manner. One general strategy is to generate a “biomolecular spectral fingerprint” by allowing biofluid components to adsorb to a SERS substrate based on their differing binding affinities.^19^ With this strategy, the resulting SERS spectrum should display differences between a patient and a control (i.e., a healthy individual), resulting in a simple and fast method of diagnosis. However, considerable limitations of a label-free approach include inevitable interfering molecules (e.g., proteins) and nonspecific binding, resulting in poor reproducibility across samples. Wang and co-workers addressed these limitations by reporting a three-step method to overcome signal interference to achieve quantitative analysis of drugs in serum.^20^ This protocol included removal of proteins from serum, enhanced drug adsorption to the SERS substrate, and the use of aggregating agents.^20^ There have been many applications of label-free SERS in clinical diagnostics, including cancer screening and diagnosis.^21−23^

Emtricitabine (FTC) is a nucleoside reverse transcriptase inhibitor (NRTI) drug, which constitutes the backbone of first-line ART and PrEP regimens and has been lauded as one of the best and most effective antiretrovirals in the market.^24^ It is often coadministered with other antiviral medications (e.g., tenofovir and integrase inhibitors).^25,26^ Previous work in our group has centered on developing an analytical SERS platform for the detection and quantification of antiviral drugs, specifically tenofovir and FTC, in aqueous matrixes.^27−29^ This previous work successfully established low detection thresholds, reaching 25 ng/mL for tenofovir and 40 ng/mL for FTC. The current study advances the efforts toward detection and quantification of FTC in human blood plasma samples by addressing several challenges in applying SERS for drug quantification in complex biological environments. A novel plasma sample pretreatment method coupled with a custom-built portable Raman scanning system for acquiring spatially resolved SERS spectra is introduced. Three quantitative methods are assessed in this paper, including conventional averaging of entire populations of SERS spectra (total population method), where all the spectra collected for each concentration were averaged, then the SERS intensity of a known FTC peak (792 cm^–1^) was used for calibrations. Another quantitative approach, the *Q*_i_ sample method, utilizes a figure-of-merit (FOM) quality index (*Q*_i_) to sample spectra from each concentration that contained the highest signal-to-noise (S/N), before proceeding with the same averaging and subtraction steps as the total population method. Finally, the distribution of all *Q*_i_ values for each concentration were modeled using cumulative distribution functions (CDFs) and used for calibrations (CDF method). The advantages of both the *Q*_i_ sample and CDF methods over the total population method as quantitative methods for SERS data are discussed in this paper. Finally, the findings of this study establish a framework for integrating SERS into HIV drug adherence monitoring, extending adherence monitoring capabilities to regions of the world that lack the resources for complex instrumentation and analytical capabilities.

## 2 Materials and Methods

### 2.1 Synthesis of Silver Colloidal Nanoparticles

The SERS substrate used in this study, silver colloidal nanoparticles (Ag CNPs), were prepared following a procedure previously published by Leopold and Lendl.^30^ Briefly, 0.3 mL of 1 M NaOH (Fisher Chemical) was added to 90 mL of 1.6 × 10^–3^ M hydroxylamine hydrochloride (NH_2_OH·HCl, Sigma). 10 mL of 1.0 × 10^–2^ M silver nitrate (AgNO_3_, Sigma) was then added to the mixture while stirring at room temperature at 360 rpm. 89.9% Milli-Q (Millipore) water was used as the solvent. The solution continued stirring for another 45 min then was stored in the dark at room temperature. Immediately after stirring, dynamic light scattering (DLS) and UV–visible spectroscopy experiments were performed on the synthesis. DLS and UV–visible measurements were taken prior to every experiment (see Figures S1 and S2, respectively). The average particle diameter was 70.27 nm and the λ_max_ was 440 nm.

**Figure 1:**
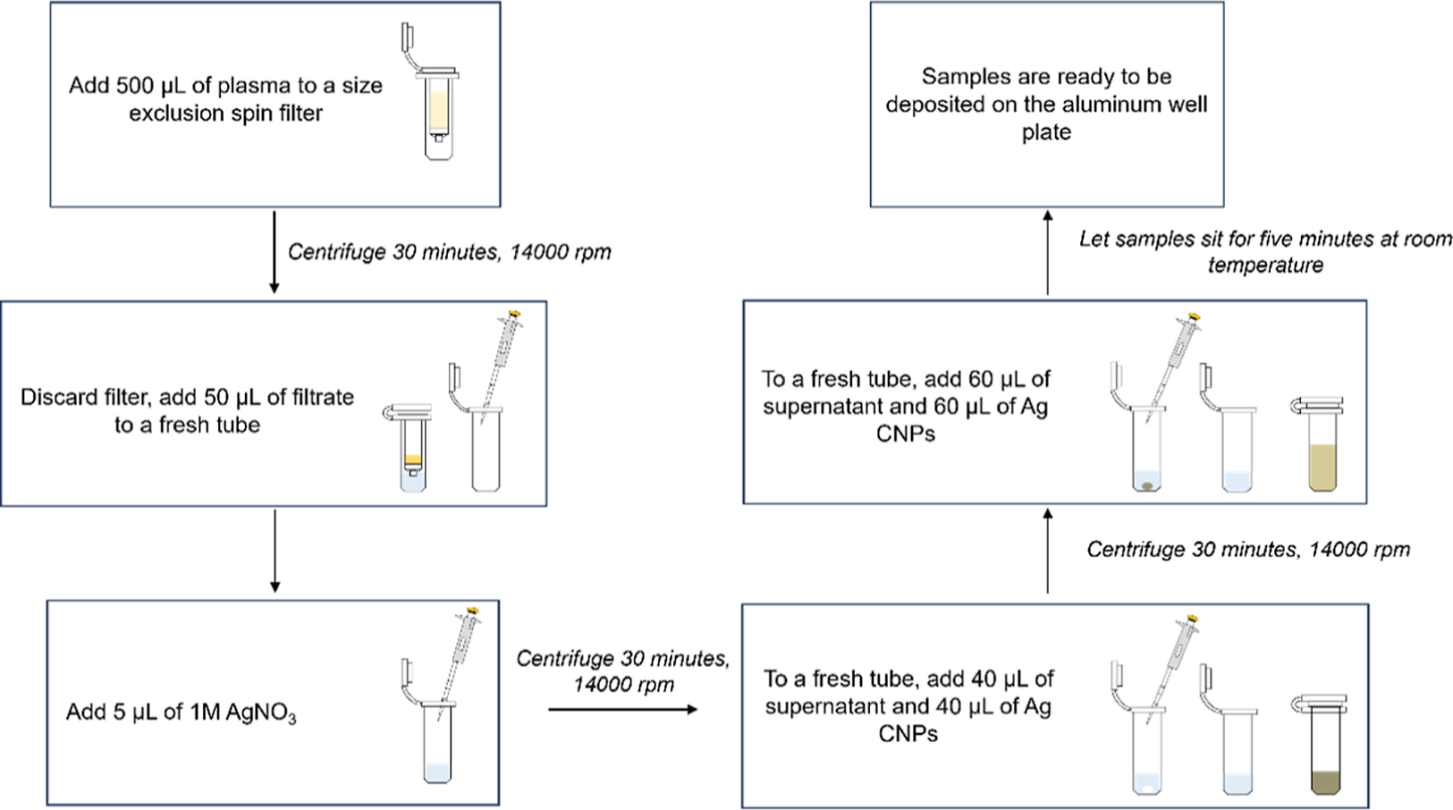
Workflow of the plasma sample treatment protocol used in this study.

**Figure 2:**
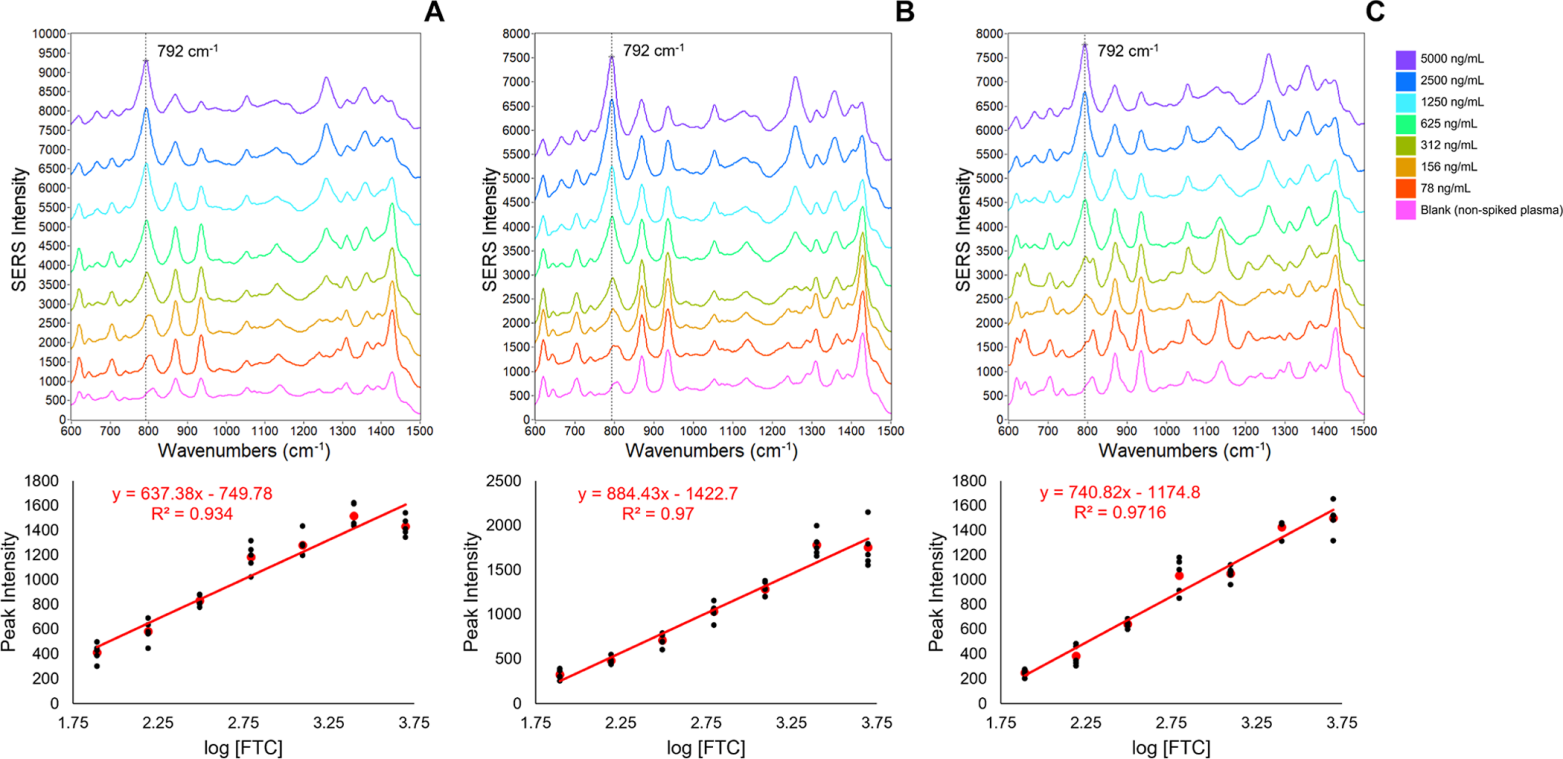
Calibration curves prepared using the total population method for three replicate experiments. (A–C) Averaged SERS spectra for all concentrations and corresponding SERS intensity calibration curves beneath. Each spectrum shown is an average of 9030 spectra (1806 spectra from each concentration replicate). Each black data point represents the difference in SERS intensities at 792 and 723 cm^–1^ for each concentration replicate. Linear regression lines were calculated using the average of all concentration replicates (red data points). The regression line, equation, and correlation coefficient for each replicate experiment are shown in red. Spectra were offset for clarity.

### 2.2 SERS Surface Preparation

An aluminum well plate constructed using certified 1100 aluminum with machined wells arranged in an 8 × 5 array was used as the SERS surface. Each well had a volume capacity of 20 μL, diameter of 6000 μm, and depth of 1778 μm. This well plate was reused for all experiments performed for this study (see Figure S3 for a photo of the aluminum well plate). The well plate was chemically cleaned using an acetic acid cleaning procedure. Briefly, the aluminum well plate was soaked for 10 min in boiling 1 M acetic acid, and cotton swabs were used to remove any loose debris in the wells on the plate. This process was repeated a total of three times, then the well plate was stored in a chemical hood until use.

### 2.3 Plasma Sample Preparation

Commercially sourced plasma from BiolVT was used is this study (female-pooled, K2 EDTA anticoagulant) and was stored at −20 °C until use. Seven 500 μL aliquots of thawed plasma were prepared and spiked (1% by volume) with an aqueous FTC solution to achieve the desired concentration gradient ranging from 5000 to 78 ng/mL. The FTC spike solutions were prepared using 89.9% Milli-Q (Millipore) water and FTC certified reference material (Millipore, source number LRAC8874). Each sample was then added to a size exclusion spin filter (Cytiva Vivaspin 500, 3 kDa molecular weight cutoff) and centrifuged for 30 min at 14,000 rpm to remove the high molecular weight plasma components (e.g., proteins). The filter containing trapped high molecular weight species was discarded and the filtrate underwent sample processing for SERS measurements.

To precipitate out Cl^–^ ions that naturally exist in human plasma,^31^ 50 μL of each filtrate was added to a fresh microcentrifuge tube and 5 μL of 1 M AgNO_3_ (Sigma) was added to each tube and vortexed for approximately 3 s. The removal of naturally occurring Cl^–^ ions in plasma is a crucial step in the sample pretreatment protocol (see Figure 1) because the removal of these ions reduces an immediate aggregation effect upon the addition of Ag CNPs^30^ that would otherwise suppress SERS signals due to hindered analyte-SERS substrate binding events. Upon addition of the AgNO_3_, an immediate AgCl white precipitate was observed in all samples. These mixtures were centrifuged for 30 min at 14,000 rpm. Next, 40 μL of the resulting supernatant from each sample was removed and added to a fresh microcentrifuge tube containing 40 μL of Ag CNPs. These samples were vortexed for approximately 5 s then centrifuged for 30 min at 14,000 rpm to facilitate the removal of any residual AgNO_3_ in the supernatants. The removal of residual AgNO_3_ is important prior to sample deposition because the presence of excess silver has the propensity to participate in redox reactions with other naturally occurring reducing agents in plasma (e.g., ascorbic acid and glutathione),^32^ resulting in greater variability in silver nanoparticle formation and subsequent SERS activity. The Ag CNP suspension contains NH_2_OH·HCl in a slight excess. Therefore, the addition of this suspension followed by centrifuging has been hypothesized to be an effective means of precipitating excess silver out of the sample through an in situ reduction reaction with the excess NH_2_OH·HCl.^30^ After centrifuging, 60 μL of the resulting supernatant from each sample was added to a fresh tube containing 60 μL of Ag CNPs. Subsequently, these samples were vortexed for approximately 5 s then allowed to sit at room temperature for approximately 5 min to allow temperature equilibration. An illustration of this process is shown in Figure 1. The samples were then deposited onto the aluminum plate, where one aluminum well held a 20 μL aliquot of a given sample. Each sample was deposited in five wells. The aluminum plate then remained in a fume hood to dry prior to acquiring SERS measurements. The remaining untreated filtrates were stored at 7 °C until needed again.

### 2.4 Instrumentation

A Raman scanning device constructed in-house was used to acquire spatially resolved SERS measurements. A Wasatch Photonics 785 nm Raman spectrometer was housed on a computer-controlled stage built using products from ThorLabs. A raster scan pattern was used for spectral acquisition of each well on the aluminum plate (42 lines in the raster pattern, 43 spectra acquired per line, and 100 μm between lines). This scan pattern acquired 1806 spectra for each well, totaling 72,240 spectra for the entire plate. All spectra were acquired using an integration time of 800 ms and 15 mW of laser power.

### 2.5 Quality Index (*Q*_i_) Calculations

A quality index (*Q*_i_) was calculated for each acquired SERS spectrum using eq 1.
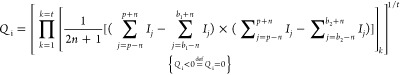
1

The *Q*_i_ is calculated directly using the SERS intensities of a peak of interest. Briefly, as shown in eq 1, the SERS intensities at wavenumbers *I*_*j*_ were summed to determine the average intensity about each peak *p* and baselines *b*_1_ and *b*_2_ (+) and (−) *n* number of wavenumbers. This summation was then raised to the inverse power of the number of peaks used to calculate the *Q*_i_, *t*. Any *Q*_i_ values <0 were defined as 0.^27,28^ For all *Q*_i_ calculations in this study, *p* was defined as 792 cm^–1^ and both *b*_1_ and *b*_2_ were defined as 723 cm^–1^. The 792 cm^–1^ peak has been shown to be a quantifiable FTC SERS peak in aqueous systems^29,33^ due to its dominant intensity compared to other peaks and was therefore used for *Q*_i_ calculations in this study. The *b*_1_ and *b*_2_ were selected based on a visual trough of the peak observed at 792 cm^–1^. See Figure S4 for a schematic representation of calculating a *Q*_i_.

### 2.6 Cumulative Distribution Function Calculations

A CDF for each FTC concentration was constructed using the spectra *Q*_i_ values (eq 1). CDFs are integral to representing the probability that a variable will take on a value less than or equal to a specific threshold, allowing the visualization of cumulative probabilities across a range. Unlike probability density functions, which focus on the likelihood of specific outcomes, CDFs accumulate these probabilities incrementally. As the *x*-values increase along the CDF, the curve rises, reflecting the increasing total probability of encountering a value up to that point.^27^ In the context of this work, the CDFs provide a visual representation of the *Q*_i_ distribution across concentrations, reflecting the changes in signal intensity of the 792 cm^–1^ peak.

The process of calculating CDFs based on the *Q*_i_ values has been described meticulously elsewhere.^27,29^ Briefly, the nonzero *Q*_i_ values (eq 1) for each sample replicate (one well on the aluminum well plate) were sorted in ascending order, where an index value of one was assigned to the spectrum corresponding to the lowest *Q*_i_ value, then increased incrementally. The response, defined as the probability, of each *Q*_i_ in the CDF was calculated by dividing each index by the highest index value. These values were then plotted as a function of the logarithm of the *Q*_i_ value corresponding to that index. A model CDF of each FTC concentration was calculated using the *Q*_i_ values from all replicates. After adding the *Q*_i_ values from all replicates of a given concentration to a single array, the same sorting, indexing, and dividing steps were performed. Therefore, the replicate CDFs visualized the *Q*_i_ distribution of a single aluminum well and the model CDFs visualized the *Q*_i_ distribution of an entire concentration. See Figure S5 for a process diagram of the CDF calculation workflow.

Calibration curves using the CDF method were generated from the model CDFs by summing the errors for each concentration (eq 2), which was calculated using all the probability points of the CDFs and defined as Σ ΔQCDF. Because these errors were pointwise differences, 500 fit points (generated from a fourth-order polynomial fit for each model CDF) spaced evenly across the CDF probability range were used to populate each model CDF at the same probability points for Σ ΔQCDF calculations.
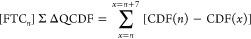
2As shown in eq 2, *n* is the index of a given concentration for notation purposes during calculations (e.g., the highest concentration is assigned an index of 0, and the blank is assigned an index of 7).

## 3 Results

### 3.1 Calibration Curves

#### 3.1.1 Total Population Method

Calibration curves using the total population method were generated by averaging all acquired spectra from each population (an aluminum well). Because each FTC concentration was deposited in five aluminum wells, five SERS spectra were obtained for each concentration. Using these spectra, the calibration curve responses were calculated by subtracting the SERS intensity at 723 cm^–1^ from that at 792 cm^–1^. These differences, reflecting the trough at 723 cm^–1^ relative to the 792 cm^–1^ FTC peak, were plotted against the logarithm of FTC concentration (Figure 2). A linear regression was then applied to the average differences at each concentration (Figure 2).

#### 3.1.2 *Q*_i_ Sample Method

**Figure 3:**
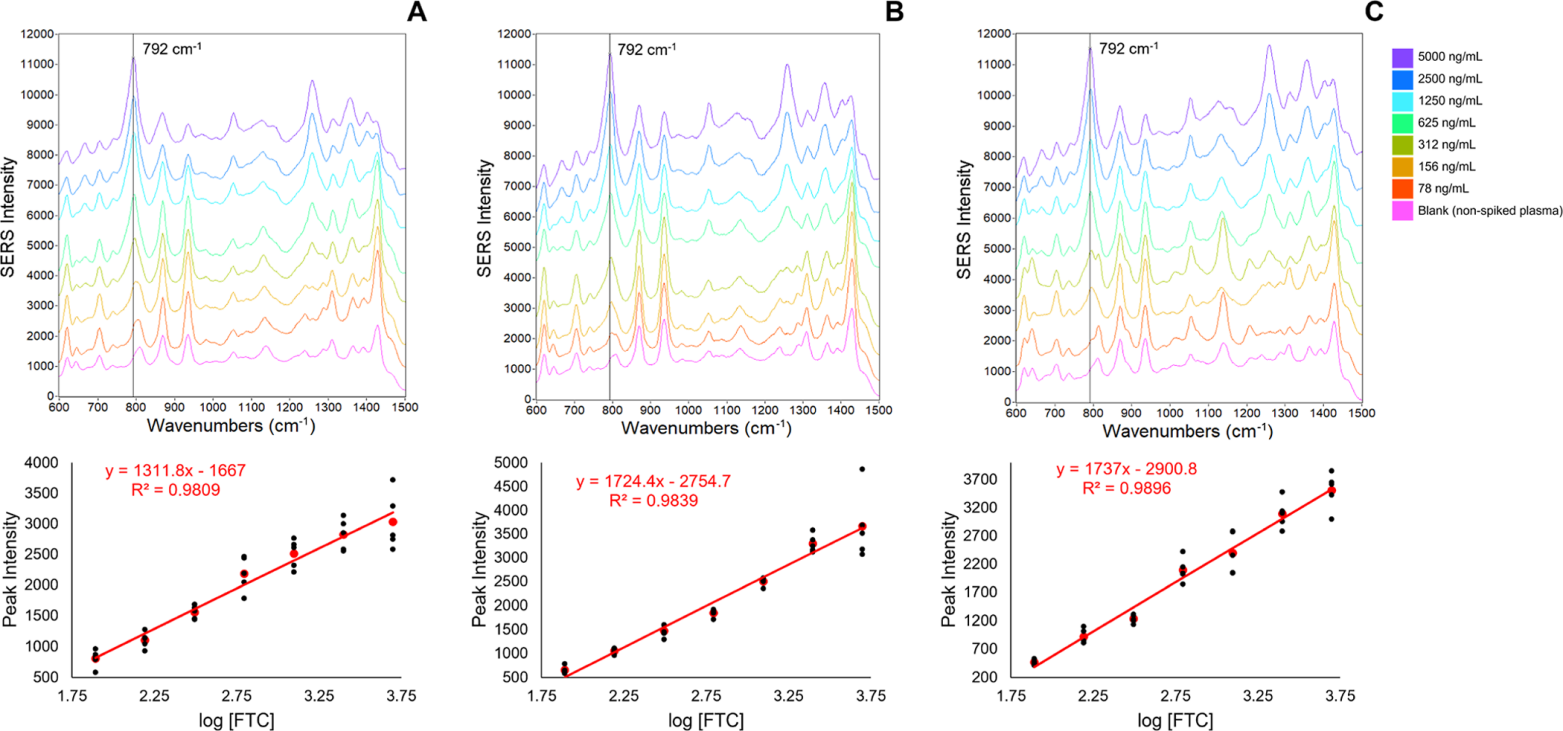
Calibration curves prepared using the *Q*_i_ sample method for three replicate experiments. (A–C) Averaged SERS spectra for all concentrations and corresponding SERS intensity calibration curves beneath. Each spectrum shown is an average of 100 spectra (20 spectra from each replicate corresponding to the highest 792 cm^–1^*Q*_i_). Each black data point represents the difference in SERS intensities at 792 and 723 cm^–1^ for each concentration replicate. Linear regression lines were calculated using the average of all concentration replicates (red data points). The regression line, equation, and correlation coefficient for each replicate experiment are shown in red. Spectra were offset for clarity.

Calibration curves using the *Q*_i_ sample method were prepared by first calculating a *Q*_i_ for all collected spectra using eq 1. The *Q*_i_ values were used as a sampling criterion to extract the spectra containing the highest S/N in each population. From each population, the spectra corresponding to the top 20 *Q*_i_ values were extracted and averaged, providing five SERS spectra for each concentration. The calibration curve responses were then calculated following the same subtraction and linear regression procedures as the total population method (Figure 3). Nonspecific plasma signals did not impact the calibration curves prepared using the total population and *Q*_i_ sample methods (Figures 2 and 3, respectively). This was demonstrated by consistent linear slopes and *R*^2^ values (shown in Figures 2 and 3) after subtracting the matrix blank SERS spectrum from the analyte spectra and repeating these analyses (Figures S6 and S7).

#### 3.1.3 CDF Method

**Figure 4:**
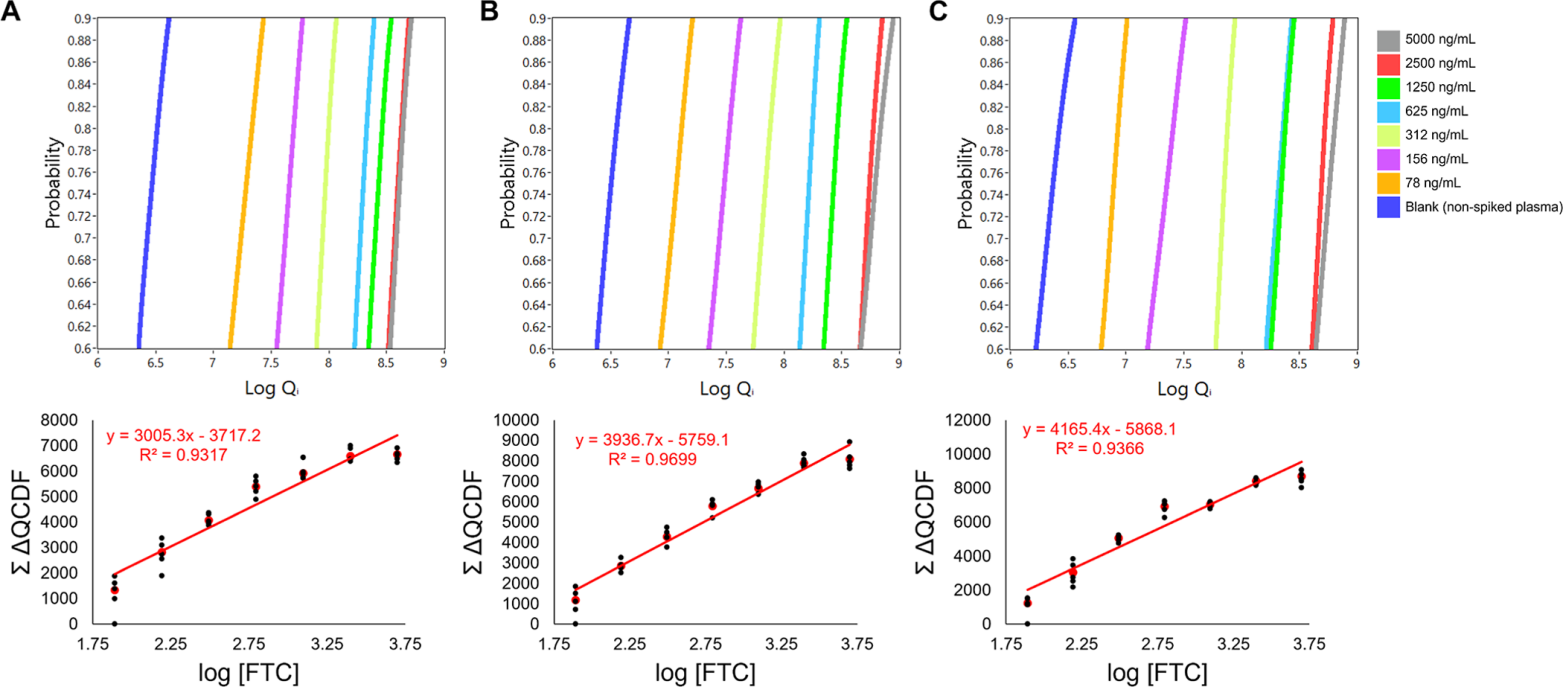
Calibration curves prepared using the CDF method for three replicate experiments. (A–C) Model CDFs of each FTC concentration and corresponding calibration curves beneath. The CDFs were constructed based on the *Q*_i_ of the 792 cm^–1^ spectral region. A 4th order polynomial was fitted to each CDF in the probability range of 0.6–0.9. The Σ ΔQCDF was calculated for each concentration (see eq 2) and plotted as a function of the logarithm of FTC concentration. The Σ ΔQCDF values of the model CDFs (red data points) were used for linear regression. Black data points represent the Σ ΔQCDF of concentration replicates. Each data point in the calibration curves was increased by the absolute value of the smallest data point, ensuring all values are positive and maintain the same intervals between points. The regression line, equation, and correlation coefficient for each replicate experiment are shown in red.

Calibration curves generated using the CDF method are shown in Figure 4. As shown in Figure 4, the CDF method calibration curves (see Section 2.6) were generated using a truncated probability range of the CDF (0.6 to 0.9). Previous studies have shown that using a narrower range of probabilities for the Σ ΔQCDF calculations provides the most meaningful information and is also most accurately fit with a polynomial function.^27,29^ The unfitted CDFs of each data set without the probability range truncation are shown in Figure S8.

### 3.2 Comparisons Between the Methods of Quantification

The average linear slope and *R*^2^ value for each of the three methods of quantification is shown in Table 1. Shown by the shaded cells of Table 1, the CDF method had the largest analytical sensitivity (represented by the linear slope), and the *Q*_i_ sample method had the greatest linearity (represented by *R*^2^).

**Table 1:** Slope and *R*^2^ Values from Calibration Curves Obtained from the Three Methods of Quantification (See Section 3.1)^a^

	quantitative method		
	total population	*Q*_i_ sample	CDF
slope	754.21	1591.05	**3702.47**
*R*^2^	0.97	**0.99**	0.95

a All slope and *R*^2^ values shown are an average of the slope and *R*^2^ values of each of the three replicate experiments (Figures 2–4). The cells corresponding to the highest *R*^2^ and slope are highlighted in bold.

Table 2 shows the relative standard deviation (RSD) for each concentration and Table 3 shows the S/N at each concentration for the three methods of quantification.

**Table 2:** RSD Values of Each Concentration for the Three Methods of Quantification^a^

	quantitative method		
concentration (ng/mL)	total population	*Q*_i_ sample	CDF
5000	0.09	0.15	**0.05**
2500	0.06	0.08	**0.03**
1250	0.07	0.09	**0.04**
625	0.11	0.09	**0.06**
312	0.07	0.07	**0.06**
156	0.15	**0.10**	0.17
78	0.16	**0.13**	0.56

a For each method, the quantitative information of the three replicate experiments (see Figures 2–4) were averaged prior to RSD calculations. The RSD values were calculated by dividing the average response of all concentration replicates by the standard deviation. The cells corresponding to the lowest RSD of each concentration are highlighted in bold.

**Table 3:** S/N Values of Each Concentration for the Three Methods of Quantification^a^

	quantitative method		
concentration (ng/mL)	total population	*Q*_i_ sample	CDF
5000	10.7	6.8	**20.8**
2500	16.2	13.2	**31.5**
1250	14.9	10.7	**25.5**
625	9.1	10.6	**16.6**
312	14.5	13.9	**17.4**
156	6.7	**9.7**	5.7
78	6.2	**7.4**	1.8

a For each method, the quantitative information of the three replicate experiments (see Figures 2–4) were averaged prior to S/N calculations. The S/N values were calculated by computing the reciprocal of the concentration RSD values shown in Table 2. The cells corresponding to the highest S/N of each concentration are highlighted in bold.

As shown by the shaded cells of Table 2, the CDF method exhibited the lowest RSD for the top five concentrations (5000–312 ng/mL) and the *Q*_i_ sample method exhibited the lowest RSD for concentrations 156 and 78 ng/mL. The CDF method had the highest S/N for concentrations (5000–312 ng/mL) but the lowest for concentrations 156 and 78 ng/mL (Table 3). The *Q*_i_ sample method had the highest S/N for the two lowest concentrations.

As shown in Table 2, the RSDs of the *Q*_i_ sample method were higher than those of the total population method for concentrations 5000, 2500, and 1250 ng/mL. The *Q*_i_ sample method sampled a small percentage of spectra corresponding to the highest *Q*_i_ values from each population before averaging, making the average more sensitive to fluctuations in SERS intensities. However, as shown in Table 2, at the four lowest concentrations (625–78 ng/mL) the opposite trend is observed, where the total population method RSD values are either greater than or equal to the RSD values of the *Q*_i_ sample method. The total population method is the only method using all spectra for calibrations, whose SERS intensities span the entire range (e.g., 0 to the maximum intensity recorded). At lower concentrations, there are fewer spectra containing analyte signal compared to higher concentrations. When averaging spectra across the entire surface in the total population method, the few spectra with analyte signal, though low in intensity, are averaged with significantly more spectra that lack any analyte signal and are comprised of random noise. By using *Q*_i_ to selectively sample spectra (*Q*_i_ sample method) with the highest analyte signal, the RSD is lower because the averaging is no longer impacted by spectra without analyte signal and randomly fluctuating noise, and the magnitude of SERS intensities is more consistent. As shown in Table 2, while the *Q*_i_ sample method is less precise at higher concentrations compared to the total population method, it doubles the analytical sensitivity as shown by the linear slopes in Table 1, enhancing the ability to distinguish between different concentrations. Importantly, as the concentration approaches the LOQ (78 ng/mL), the *Q*_i_ sample method is superior to both the total population and CDF methods in terms of S/N (Table 3).

The CDF method (Table 2) exhibited the lowest RSD values for the top five concentrations (5000–312 ng/mL). However, this method also exhibited the highest RSD (and lowest S/N) for the two lowest concentrations (156 and 78 ng/mL). As the concentration decreases, more poor S/N spectra comprise the CDFs, which raises the RSD due to the decreasing S/N, as shown in Table 3. By incorporating all the meaningful population data (i.e., all spectra that have a nonzero *Q*_i_) into the CDFs shown in Figure 4, preparing calibration curves following the CDF method achieves an optimal balance: the response values are no longer diminished by averaging (as observed in the total population method), and the RSD at higher concentrations remains manageable (unlike the elevated RSDs observed in the *Q*_i_ sample method). The CDF approach allows for the inclusion of all the meaningful spectral information on each population without compromising analytical sensitivity or S/N, in contrast to the limitations observed when only averaging spectra. Despite a reduced precision (Table 2) and S/N (Table 3) at lower concentrations, there is a significant increase in analytical sensitivity, as indicated by the differences in linear slopes between the CDF and *Q*_i_ sample methods (Table 1).

To demonstrate the importance of using *Q*_i_ as a metric for constructing the CDFs, a quantitative analysis following the CDF method was performed using only the SERS intensity at 792 cm^–1^ to construct the CDFs (data shown in Figure S9). The calibration curves generated using CDFs of the SERS intensity at 792 cm^–1^ (Figure S9) exhibited considerably higher replicate standard deviations and decreased linearity compared to the calibration curves generated from CDFs of *Q*_i_ values (Figure 4). These observations highlight the impact of the congested plasma and SERS background, where relying on raw SERS intensities introduces extraneous information into the quantitative analysis and distorts the results. Calculating a *Q*_i_ for each spectrum and using this metric to construct CDFs addresses this issue, where the SERS intensity of the 792 cm^–1^ peak is used to calculate the *Q*_i_, and the interfering background is removed by subtracting the SERS intensity of the peak baseline (see eq 1 and Section 2.5).

## 4 Discussion

**Figure 5:**
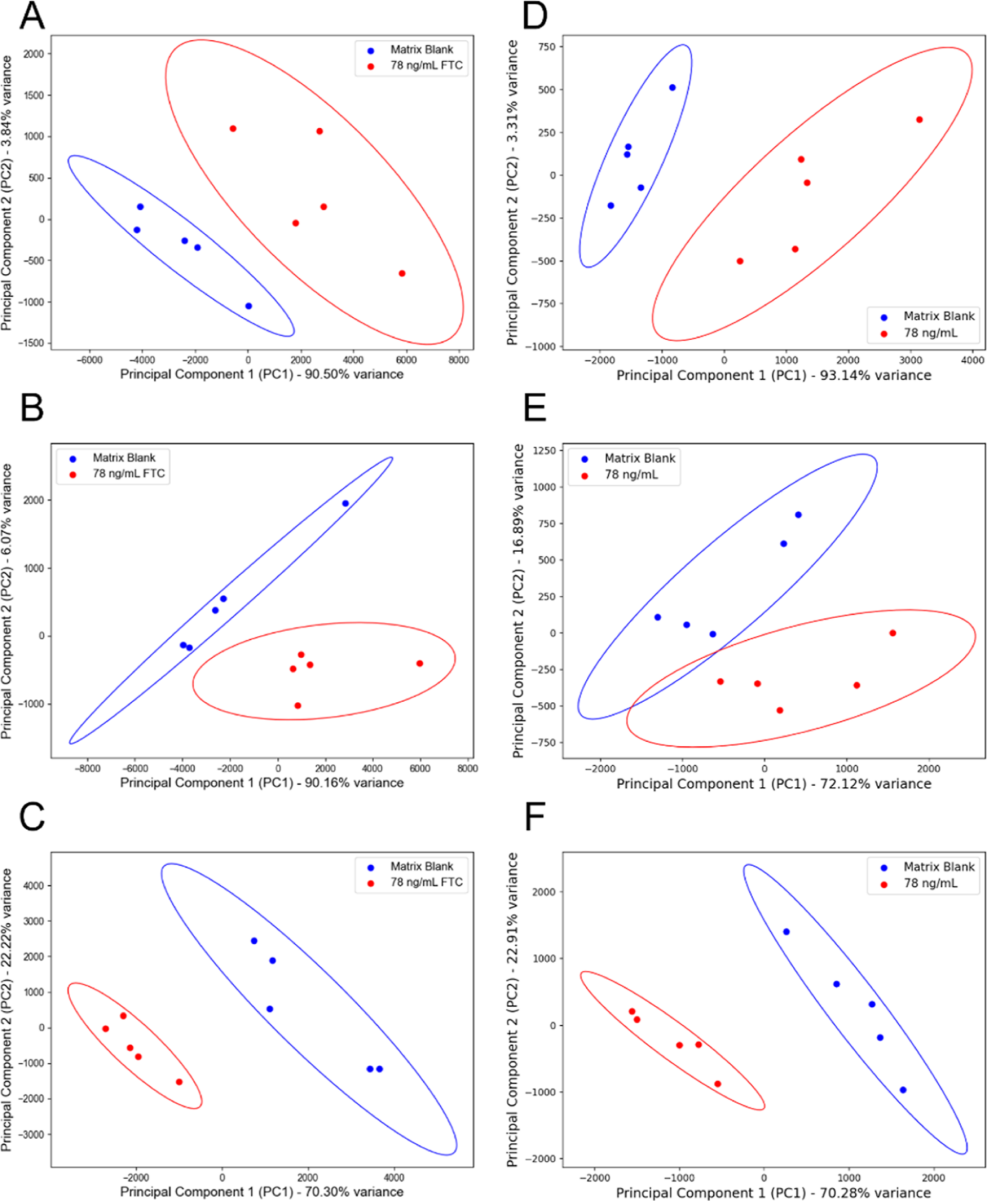
PCA of the matrix blank (blue) and 78 ng/mL (red) SERS spectra used for calibrations in the (A–C) *Q*_i_ sample method and (D–F) total population method. The Python library sklearn was used for PCA.^35^ The spectra were preprocessed by first truncating the spectral region (585.48 to 1710.01 cm^–1^) followed by applying an improved asymmetrically reweighted penalized least-squares (IarPLS) background correction algorithm^36,37^ (see Figure S10). The first two PC scores were plotted against each other, and the explained variance ratios for each PC are shown on corresponding axes. A 95% confidence ellipsoid of each group is shown. A detailed description of the PCA workflow is described in Figure S11.

This study demonstrates the advantages of statistically analyzing large populations of SERS spectral through the *Q*_i_ sample and CDF methods, rather than relying on conventional averaging (the total population method). Specifically, CDF-based analysis of *Q*_i_ values (CDF method) enhances analytical sensitivity in calibration curves, as demonstrated in Figure 4 and Table 1. Alternatively, using *Q*_i_ values to exclude low S/N spectra (*Q*_i_ sample method) improves linearity (Table 1) and S/N (Table 3) at concentrations near the limit of quantification (LOQ). Figure 5 further illustrates this by showing principal component analysis (PCA) of spectra used for calibrations in both the total population and *Q*_i_ sample methods.^34^ In a replicate experiment using the total population method (Figure 5E), the 95% confidence ellipsoids of the matrix blank and 78 ng/mL SERS spectra overlap, highlighting the diminished ability to distinguish between the matrix blank and the lowest analyte concentration. In contrast, the *Q*_i_ sample method consistently shows separation of the 95% confidence ellipsoids across all experiment replicates, further supporting the effectiveness of this approach.

Histograms illustrating the distribution of the 792 cm^–1^ peak *Q*_i_ values (Figures S12–S14) highlight the heterogeneity of the solid SERS surface in terms of SERS enhancements. Collecting spatially resolved SERS spectra of the dried samples provided spectra that range from containing no analyte signal intensity to extremely strong analyte signal intensity. Because the majority of acquired spectra had little to no analyte signal (demonstrated by the histograms of *Q*_i_ values seen in Figures S12–S14), the true distribution of the analyte SERS intensities was exponential. Interestingly, the histograms shown in Figures S12–S14 depict both an exponential and pseudo normal distribution for all concentrations. These observations are divergent from previous aqueous studies^29^ that report exclusive exponential distributions across multiple concentrations of FTC. Given the complexity of a plasma matrix, this change in distribution is likely the result of interfering species with similar binding affinities to the SERS substrates that have spectral signatures close to that of the FTC signature at 792 cm^–1^.

Much is still unknown about the reactions that occur upon adding AgNO_3_ to the filtered plasma, and how this could change across patient metabolic profiles. With a working concentration of 100 mM of AgNO_3_ in the filtered plasma, it was hypothesized that this would predominantly precipitate out Cl^–^ ions that are present at a concentration of approximately 100 mEq/L in human plasma^31^ in the form of AgCl. A white pellet formation after adding the AgNO_3_ to the plasma supports this hypothesis. However, due to the abundance of reducing agents in plasma (e.g., ascorbic acid and glutathione),^32^ the possibility of other redox reactions occurring upon the addition of AgNO_3_, an oxidizer, must be considered. To remove residual AgNO_3_ from the sample, an additional centrifugation step was performed using Ag CNPs (Figure 1). The colloidal nanoparticle suspension, which contained hydroxylamine hydrochloride in slight excess, was expected to cause the silver to precipitate out of solution. This precipitate, along with the Ag CNPs, was then separated from the rest of the sample by centrifugation, resulting in a small dark brown pellet at the bottom of the tube (data not shown).

**Figure 6:**
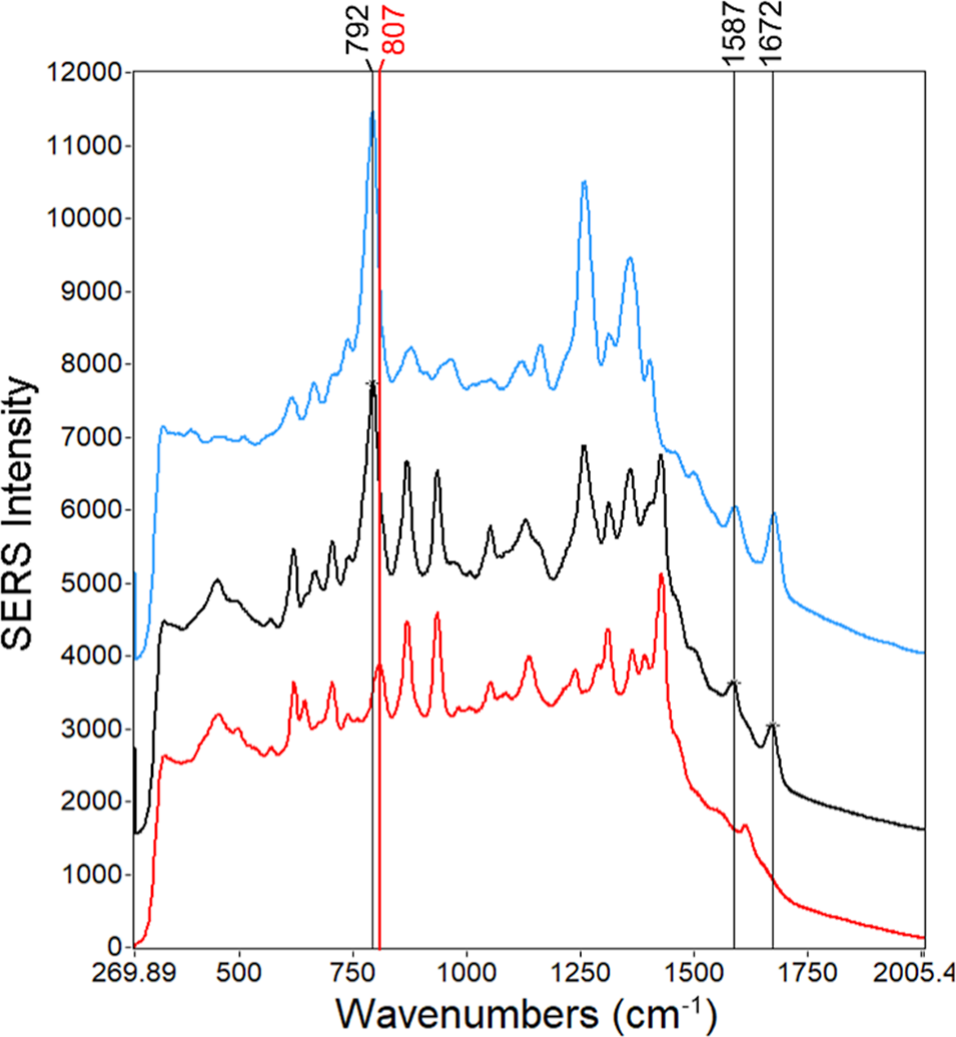
SERS spectra of aqueous 1250 ng/mL FTC (blue), plasma containing 1250 ng/mL of FTC (black), and nonspiked plasma (red). Relevant peaks from each spectrum are noted by a vertical line with their wavenumber written at the top in the color corresponding to the sample type. Each spectrum shown is an average of 25 spectra, each acquired using 15 mW of laser power and 800 ms integration time.

The results presented in this paper were produced from three independently prepared experiments, suggesting a degree of reproducibility with the sample preparation protocol (Figure 1). However, shown in Figure 6, there are still several other smaller molecular weight species exhibiting enhanced signal. This was expected, as the sample pretreatment simply consisted of size exclusion centrifuge filters, AgNO_3_, and Ag CNPs. Regardless, there are three observable peaks unique to FTC: 792 cm^–1^, ring breathing; 1586 cm^–1^, NH_2_ bending;^29^ 1672 cm^–1^, C=C, C=N, and C=O stretching.^38^ Additional peak assignments of the aqueous FTC SERS spectrum are shown in Table S1 accompanied by Figure S15. The data presented in this paper and in previous studies^29^ shows the 792 cm^–1^ peak is a consistently detectable and quantifiable spectral signature of FTC. However, Figure 6 shows a potential peak overlap between the 792 cm^–1^ peak and a peak at 807 cm^–1^, which has been reported as glutathione.^39^ Because of this, further studies are ongoing that focus on deconvolution and other complex data analysis methods to address peak overlap.

## 5 Conclusion

In this paper, a sensitive and reproducible SERS-based method to detect and quantify FTC, a common antiretroviral used for the prevention and treatment of HIV infections, is reported. The sample pretreatment method using size exclusion centrifuge filters, AgNO_3_, and Ag CNPs allows reproducible SERS-based quantification of FTC down to 78 ng/mL in human plasma. The sample pretreatment methods applied in this study sufficiently isolated FTC, in an otherwise highly complex matrix, without the use of labeling. Additionally, evaporating the samples to dryness concentrated each sample, forced aggregation of Ag CNPs, and created regions of significant SERS enhancements. Three experiment replicates were used to evaluate the reproducibility of the plasma sample treatment protocol. This study investigated three methods of quantification and found that statistically analyzing SERS spectra across an entire surface (either by sampling spectra from the population using the *Q*_i_ sample method or modeling the *Q*_i_ distribution with CDFs) yields superior results for quantitative analysis compared to the conventional approach of averaging all collected spectra (total population method). Particularly, the *Q*_i_ sample method maximizes the linearity and S/N at low concentrations (156 and 78 ng/mL). Conversely, the CDF method minimizes the RSD at higher concentrations and maximizes analytical sensitivity.

Collecting SERS spectra spatially with a Raman scanning system (described in Section 2.4) enables the use of the *Q*_i_ sample and CDF methods discussed in this study. These methods are not feasible with the standard practice of acquiring SERS data across a large surface area using a single, large laser spot size. The scanning system allows for multiple spectra to be taken across a surface with micrometer resolution, whereas spectrometers with a large laser spot size integrate all spectral information on a surface into a single spectrum. The total population method discussed in this paper represents this standard practice, where all spectra collected for each surface were averaged. The results of this study demonstrate that quantitative analysis of SERS data is improved by statistically treating spatially resolved SERS spectra across a surface (i.e., by the *Q*_i_ sample or CDF methods), compared to standard practices of averaging the spectral data of entire surfaces (total population method).

Multivariate methods are currently being explored, including PLS and multilinear regression (MLR), to enhance the unique FTC signals shown in Figure 6. Furthermore, the CDFs used in the CDF method were based on the *Q*_i_ of a single FTC peak (792 cm^–1^). Another approach to constructing CDFs using PLS regression vectors is also under investigation. Further studies are needed that focus on analyzing plasma samples from a large pool of patients to determine if quantifying FTC concentrations remains reproducible across different metabolic profiles. Data analysis methods focusing on deconvoluting the SERS spectra to isolate FTC signals could also contribute toward standardizing quantitative analysis across different metabolic profiles. Overall, the data presented in this study demonstrates the capability of SERS as an effective technique for detecting and quantifying FTC in plasma samples for drug adherence monitoring, a significant step toward widespread implementation of SERS in clinical settings.

## Supporting Information Available

DLS of nanoparticles, UV–vis of nanoparticles, image of aluminum well plate, schematic of *Q*_i_ calculation process, process diagram of CDF calculation workflow, calibration curves calculated using the total population method after subtracting the matrix blank SERS spectrum, calibration curves calculated using the *Q*_i_ sample method after subtracting the matrix blank SERS spectrum, unfitted CDFs of the entire probability range (0–1), CDF method analysis using the SERS intensity at 792 cm^–1^ to construct CDFs, preprocessed SERS spectra used for PCA, overview of PCA workflow, histograms of the *Q*_i_ distribution of all data sets, chemical structure of FTC, peak assignments of an aqueous FTC SERS spectrum (PDF)
