# Supplementary material for: Quantification of Antiretroviral Drug Emtricitabine in Human Plasma by Surface Enhanced Raman Spectroscopy
Source: ACS Omega. 2024 Nov 25;10(5):4315–25. doi: 10.1021/acsomega.4c06162 (PMC11822520; doi:10.1021/acsomega.4c06162)
Supplement: Supplementary file 1 — ao4c06162_si_001.pdf [file ao4c06162_si_001.pdf]

Supplementary Materials for

Quantification of antiretroviral drug emtricitabine (FTC) in human plasma by surface enhanced Raman spectroscopy (SERS)

Marguerite R. Butler[1], Terry A. Jacot[2], Sucharita M. Dutta[2], Gustavo F. Doncel[2], John B. Cooper[1*]

[1]Department of Chemistry and Biochemistry, Old Dominion University, Norfolk, VA 23529, USA
[2]CONRAD, Eastern Virginia Medical School, Norfolk, VA 23507, USA
[*]Corresponding author John B. Cooper, jcooper@odu.edu

This file contains the following:
Figures S1 – S15
Table S1

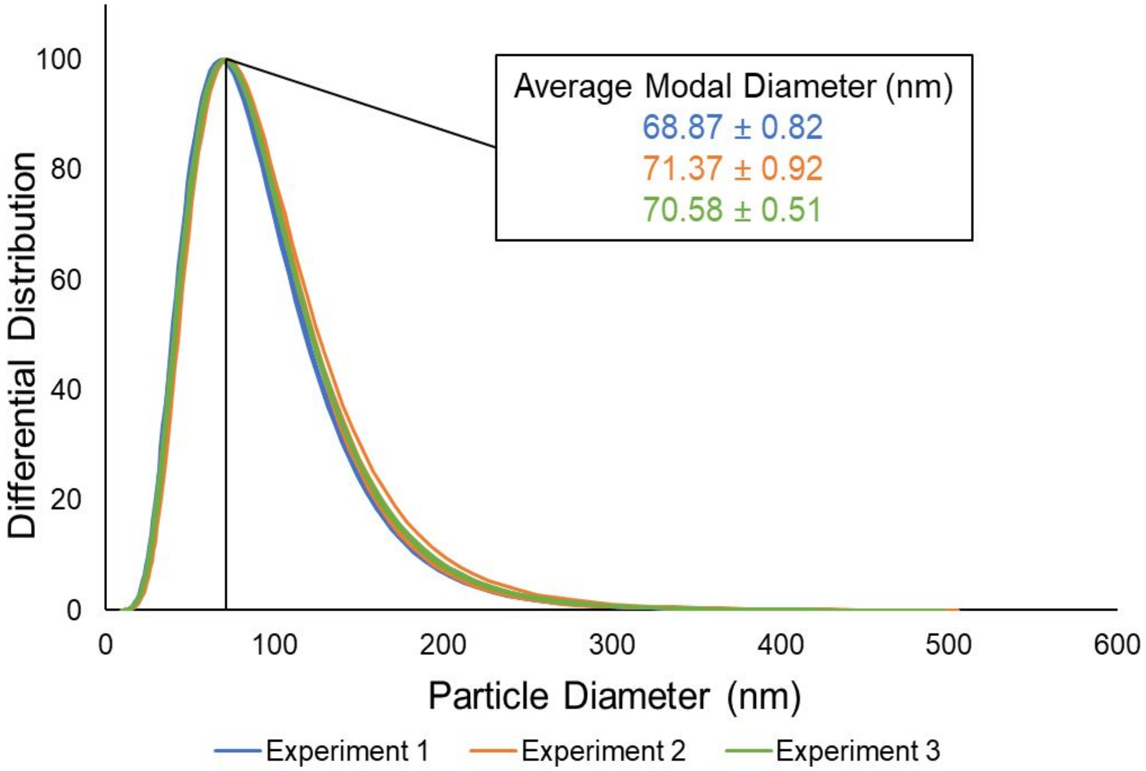

Figure S1. Differential distributions plotted as a function of particle diameter of dynamic light scattering (DLS) measurements for the Ag CNP synthesis used in this study. These measurements were taken the same day as each corresponding experiment. The average modal diameter ± the standard deviation of five trials is shown for each experiment.

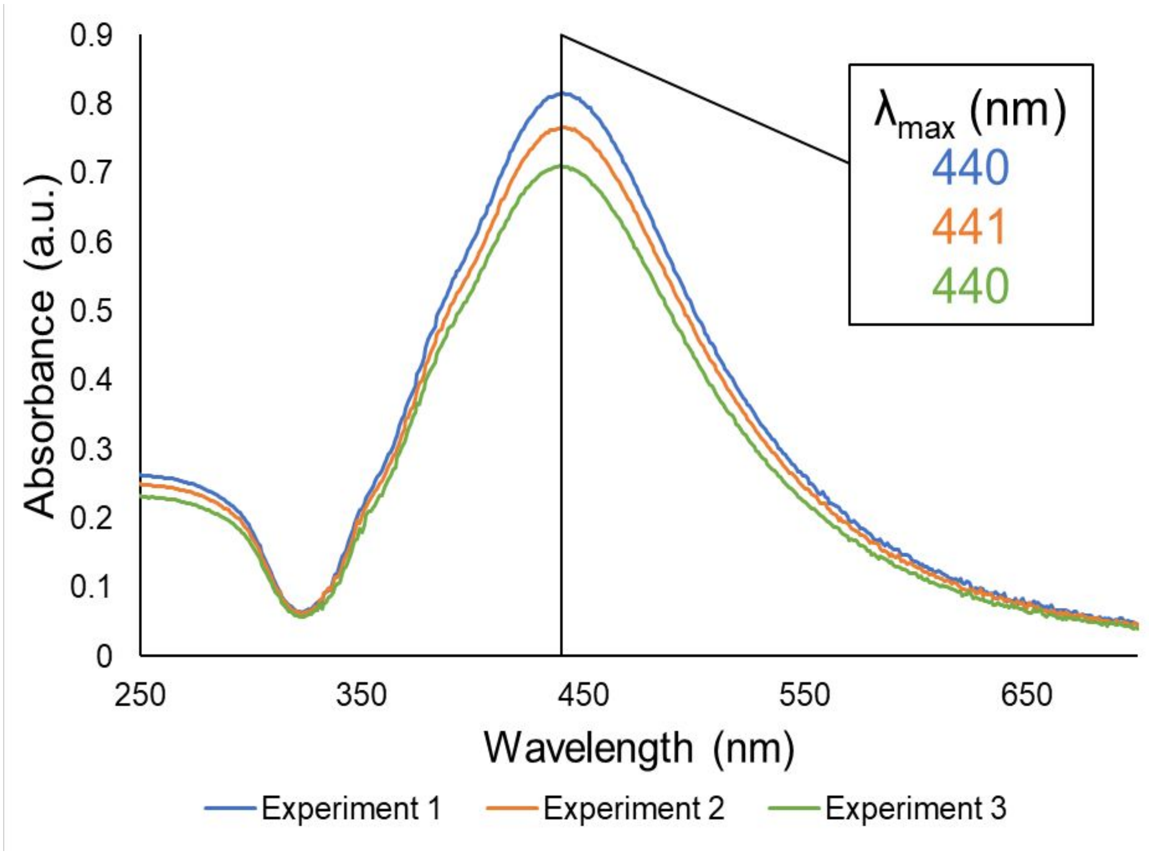

Figure S2. Ultraviolet-visible (UV-vis) spectroscopy spectra of the Ag colloidal nanoparticle (Ag CNP) synthesis used in this study. These measurements were taken the same day as each corresponding experiment.

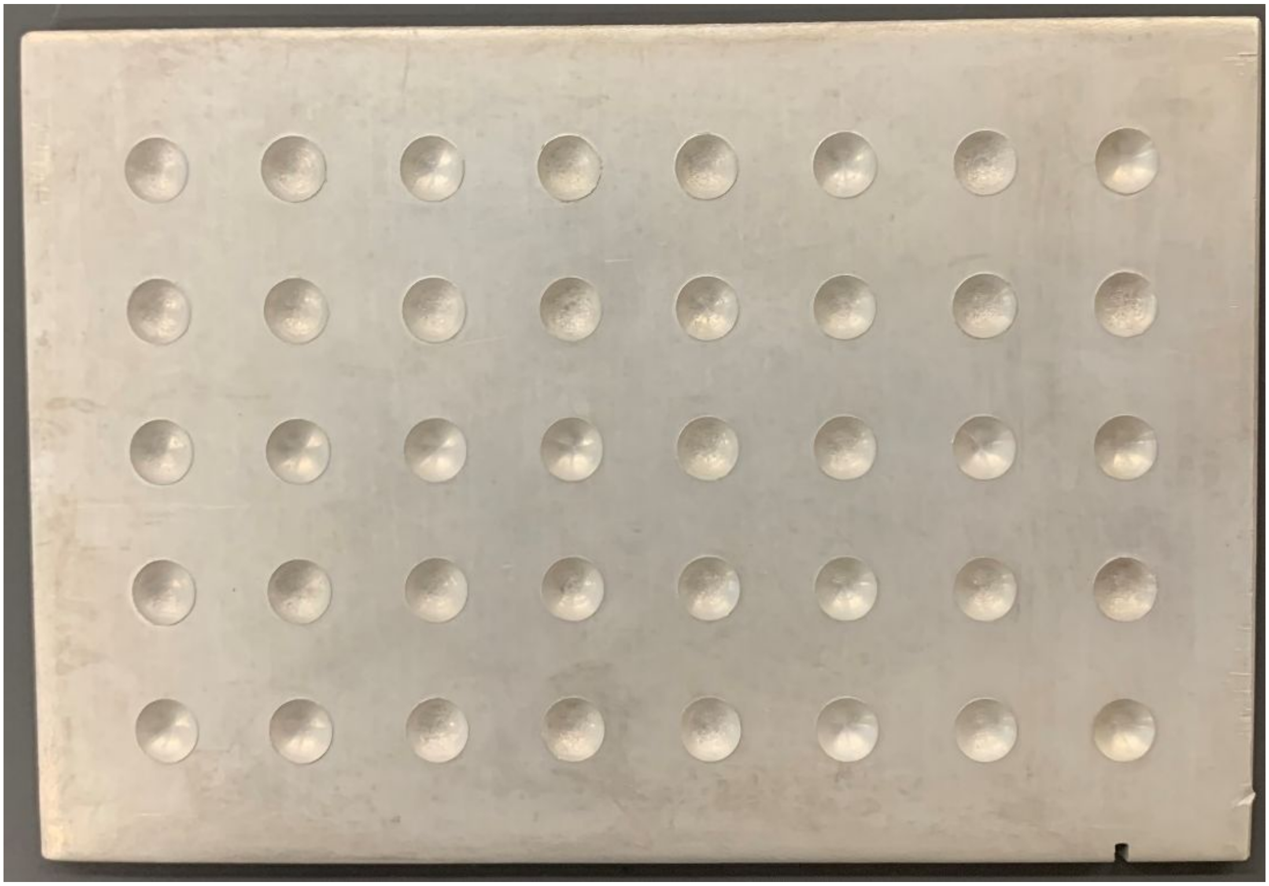

Figure S3. Image of the aluminum well plate that was used as the SERS surface.

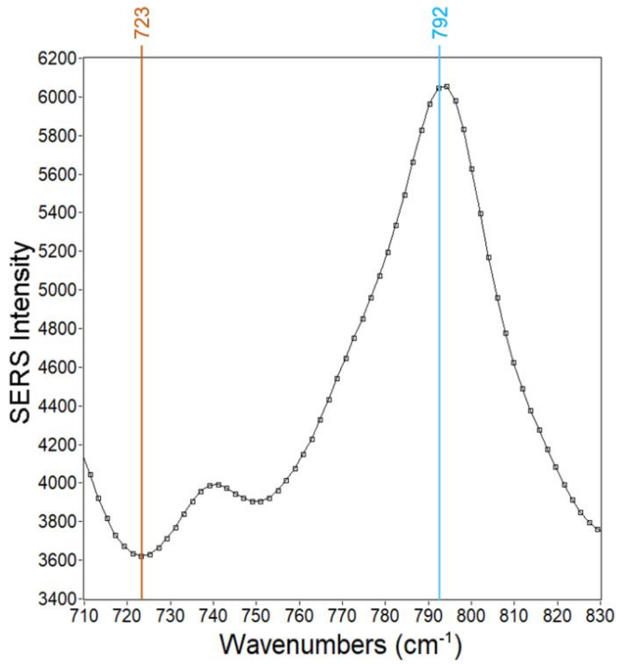

$$Q_i = \left[\prod_{k=1}^{k=t}\left[\frac{1}{2n+1}\left[\left(\sum_{j=p-n}^{p+n} I_j - \sum_{j=b_1-n}^{b_1+n} I_j\right)\times\left(\sum_{j=p-n}^{p+n} I_j - \sum_{j=b_2-n}^{b_2+n} I_j\right)\right]\right]_k\right]^{1/t}$$

$$\{Q_i<0 \stackrel{\text{def}}{=} Q_i=0\}$$

Q_i equation variable	Definition	Value (used in this study)
n	Number of wavenumbers (+) and (-) p, b_1, and b_2 wavenumber positions (represented by single data points on the spectrum)	3
p	Wavenumber position of peak	792 cm^{-1}
b_1 and b_2	Wavenumber position(s) of peak trough or baseline	723 cm^{-1}
I_j	SERS intensity at wavenumber j defined by p, b_1, b_2, and n	
t	Number of SERS peaks used for Q_i calculation	1

Figure S4. Schematic representation of the Q_i calculation process for each spectrum, where the peak and baseline wavenumbers used for calculations are shown in blue and orange, respectively. The Q_i equation (Eq. 1 in main text) and accompanying table of equation variable definitions and relevant values used for the Q_i calculations in this study are also shown.

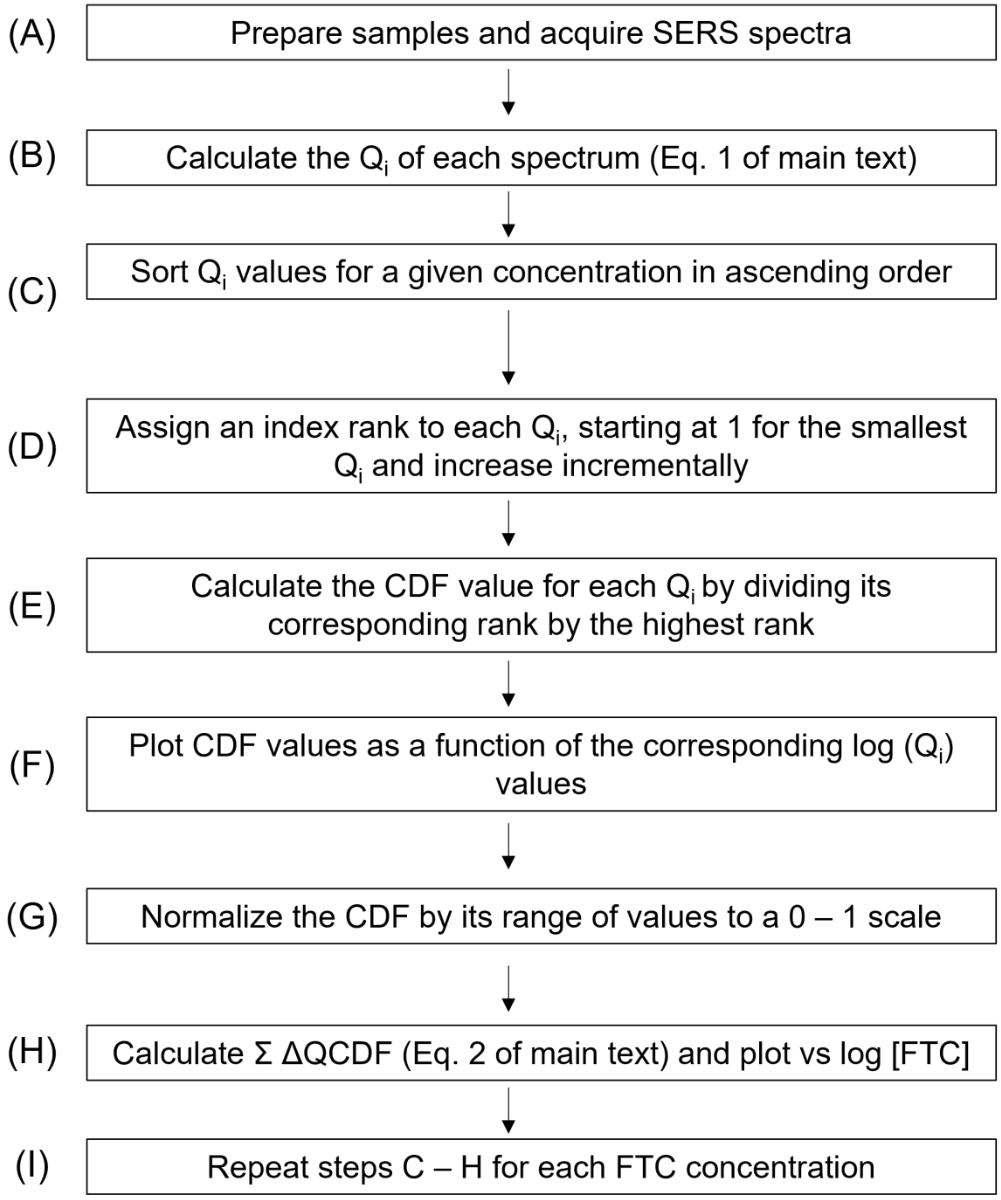

Figure S5. Process diagram of the CDF calculation workflow applied in this study.

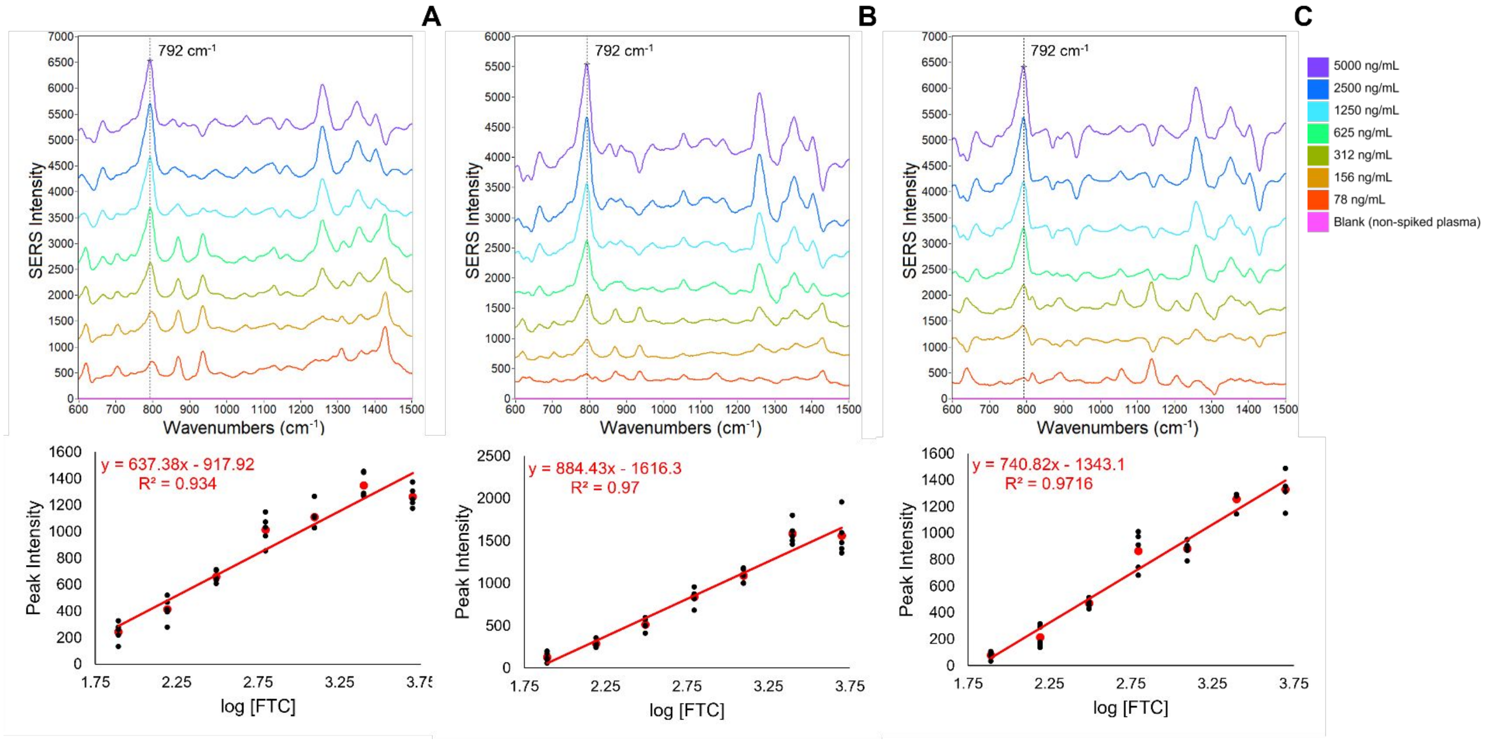

Figure S6. Calibration curves prepared by subtracting the matrix blank SERS spectrum from the analyte spectra and using the total population method for three replicate experiments. **(A – C)** Averaged SERS spectra for all concentrations after subtracting the matrix blank spectrum and corresponding SERS intensity calibration curves beneath. Each spectrum shown is an average of 9030 spectra (1806 spectra from each concentration replicate). Each black data point represents the difference in SERS intensities at 792 cm^{-1} and 723 cm^{-1} for each concentration replicate. Linear regression lines were calculated using the average of all concentration replicates (red data points). The regression line, equation, and correlation coefficient for each replicate experiment are shown in red. Spectra were offset for clarity.

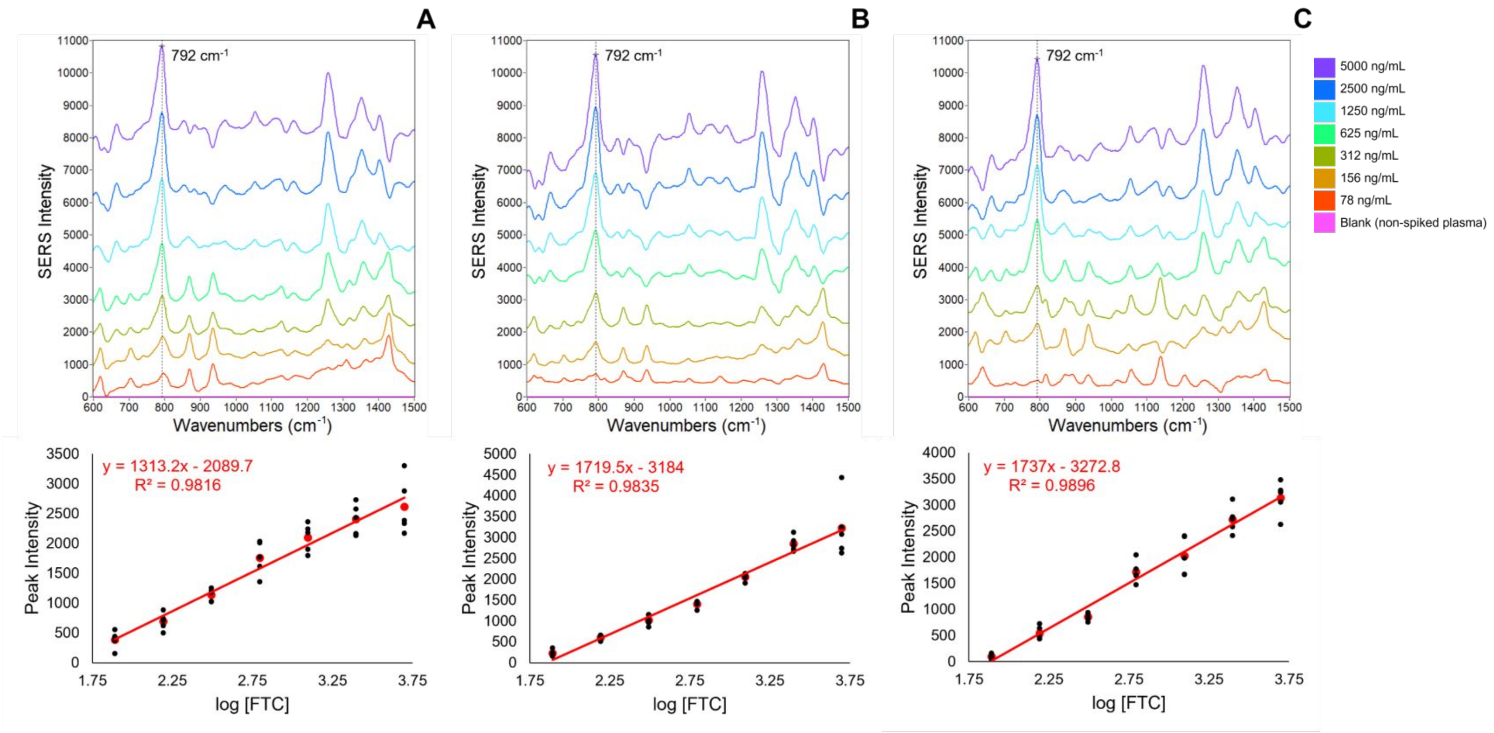

Figure S7. Calibration curves prepared by subtracting the matrix blank SERS spectrum from the analyte spectra and using the Q_i sample method for three replicate experiments. **(A – C)** Averaged SERS spectra for all concentrations after subtracting the matrix blank spectrum and corresponding SERS intensity calibration curves beneath. Each spectrum shown is an average of 100 spectra (20 spectra from each replicate corresponding to the highest 792 cm^{-1} Q_i). Each black data point represents the difference in SERS intensities at 792 cm^{-1} and 723 cm^{-1} for each concentration replicate. Linear regression lines were calculated using the average of all concentration replicates (red data points). The regression line, equation, and correlation coefficient for each replicate experiment are shown in red. Spectra were offset for clarity.

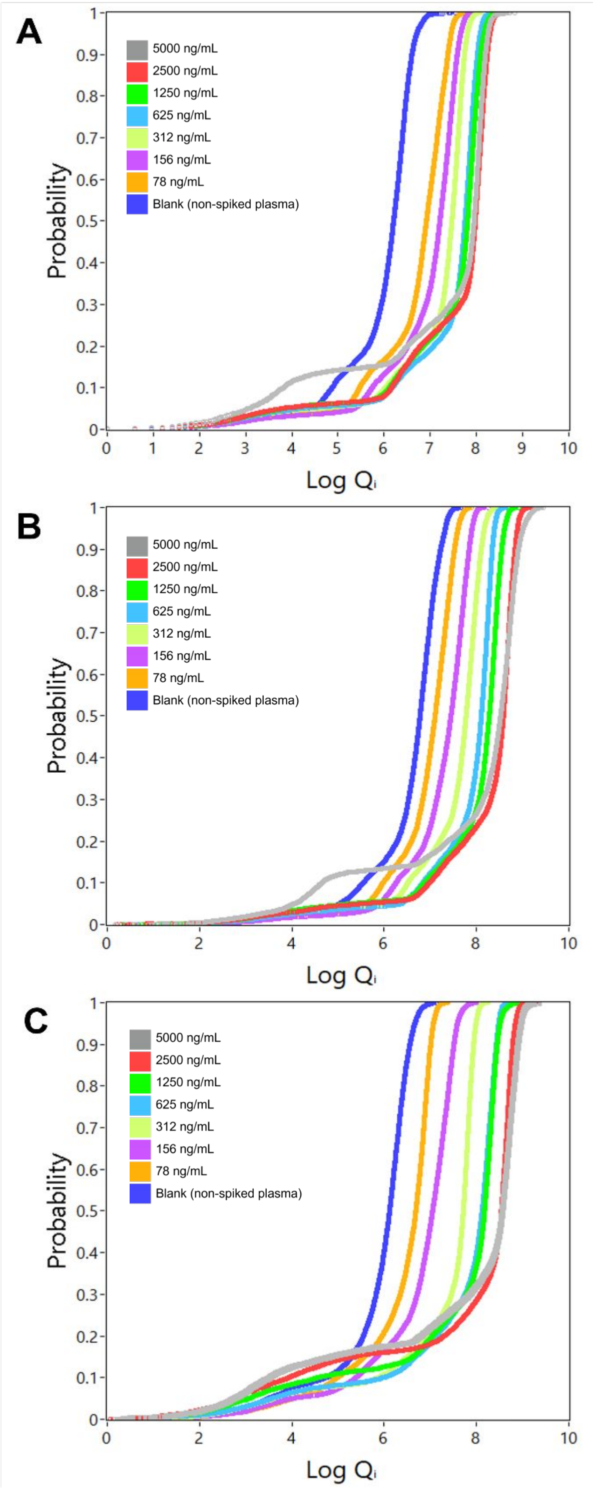

Figure S8. **(A – C)**. Raw unfitted CDFs for all concentrations for the three experiment replicates of the entire probability range (0 – 1). The CDFs were constructed based on the Q_i of the 792 cm^{-1} spectral region.

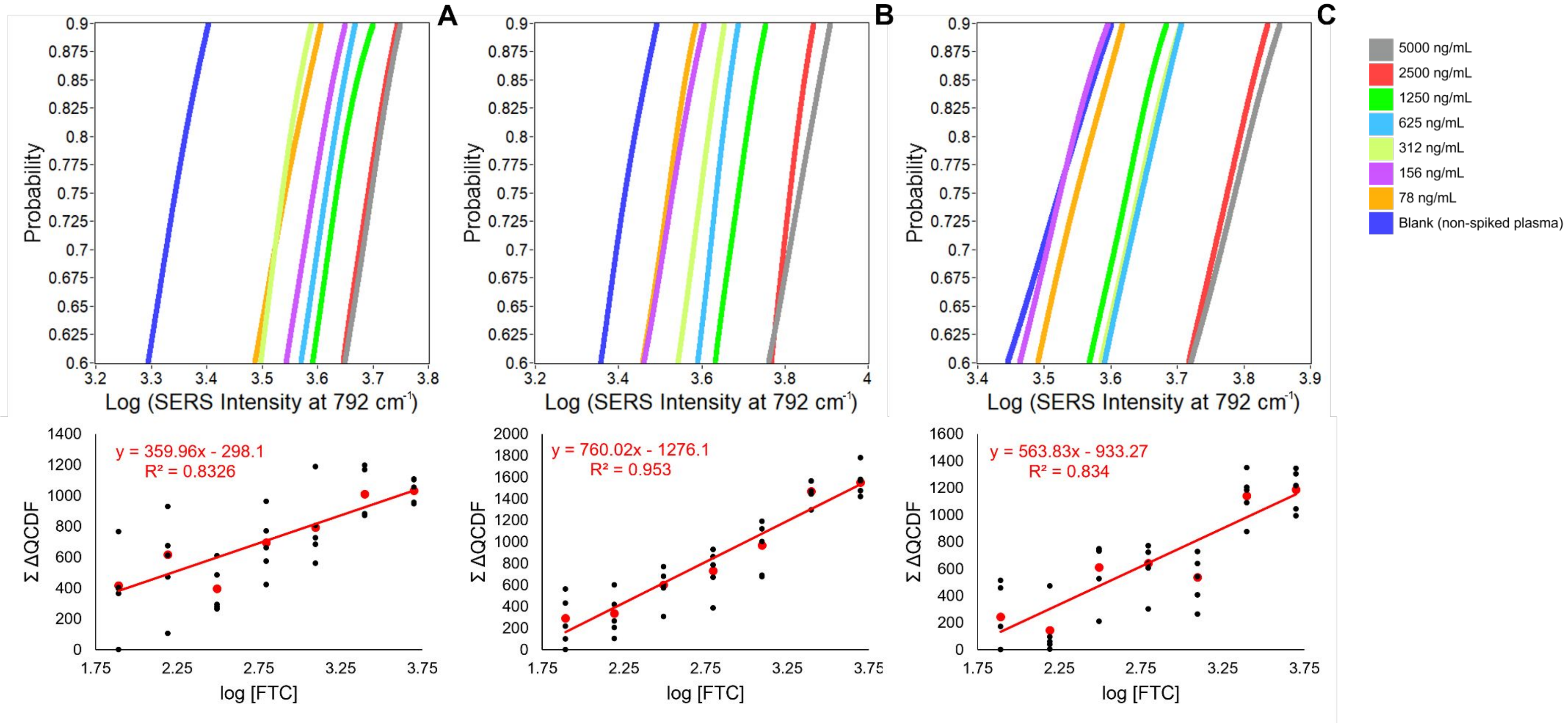

Figure S9. Calibration curves prepared using the CDF method for three replicate experiments. The CDFs were calculated using the SERS intensity at 792 cm^{-1}. **(A-C)** Model CDFs of each FTC concentration and corresponding calibration curves beneath. A 4th order polynomial was fitted to each CDF in the probability range of 0.6 – 0.9. The Σ ΔQCDF was calculated for each concentration (see Eq. 2 of the main text) and plotted as a function of the logarithm of FTC concentration. The Σ ΔQCDF values of the model CDFs (red data points) were used for linear regression. Black data points represent the Σ ΔQCDF of concentration replicates. Each data point in the calibration curves was increased by the absolute value of the smallest data point, ensuring all values are positive and maintain the same intervals between points. The regression line, equation, and correlation coefficient for each replicate experiment are shown in red.

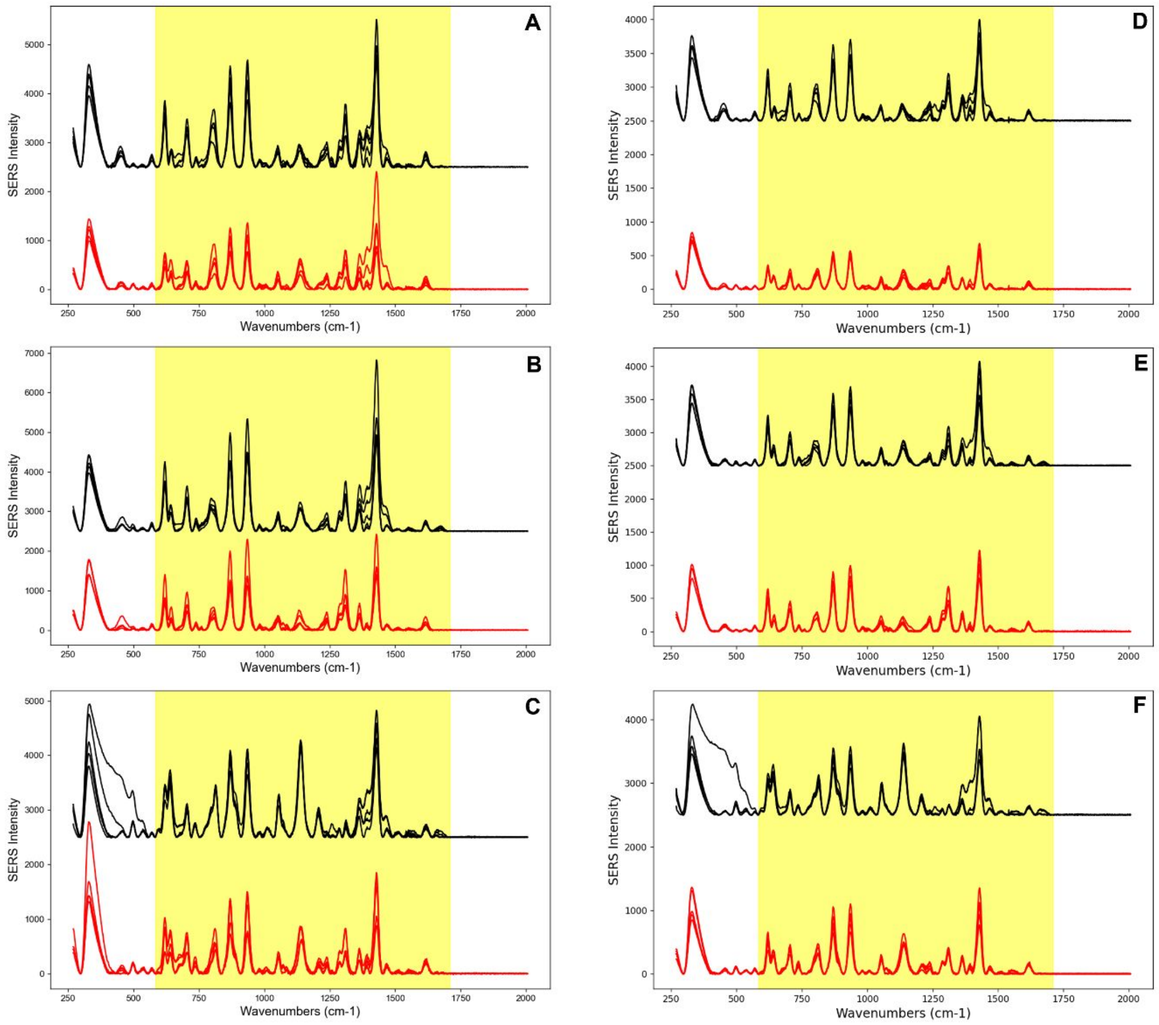

Figure S10. Preprocessed SERS spectra used for PCA from **(A – C)** the total population method and **(D – F)** the Q_i sample method of the three experiment replicates, where 78 ng/mL spectra are shown in black and matrix blank spectra shown in red. The spectral region used for PCA (585.48 cm^{-1} to 1710.01 cm^{-1}) is highlighted in yellow. Each group of spectra were offset for clarity.

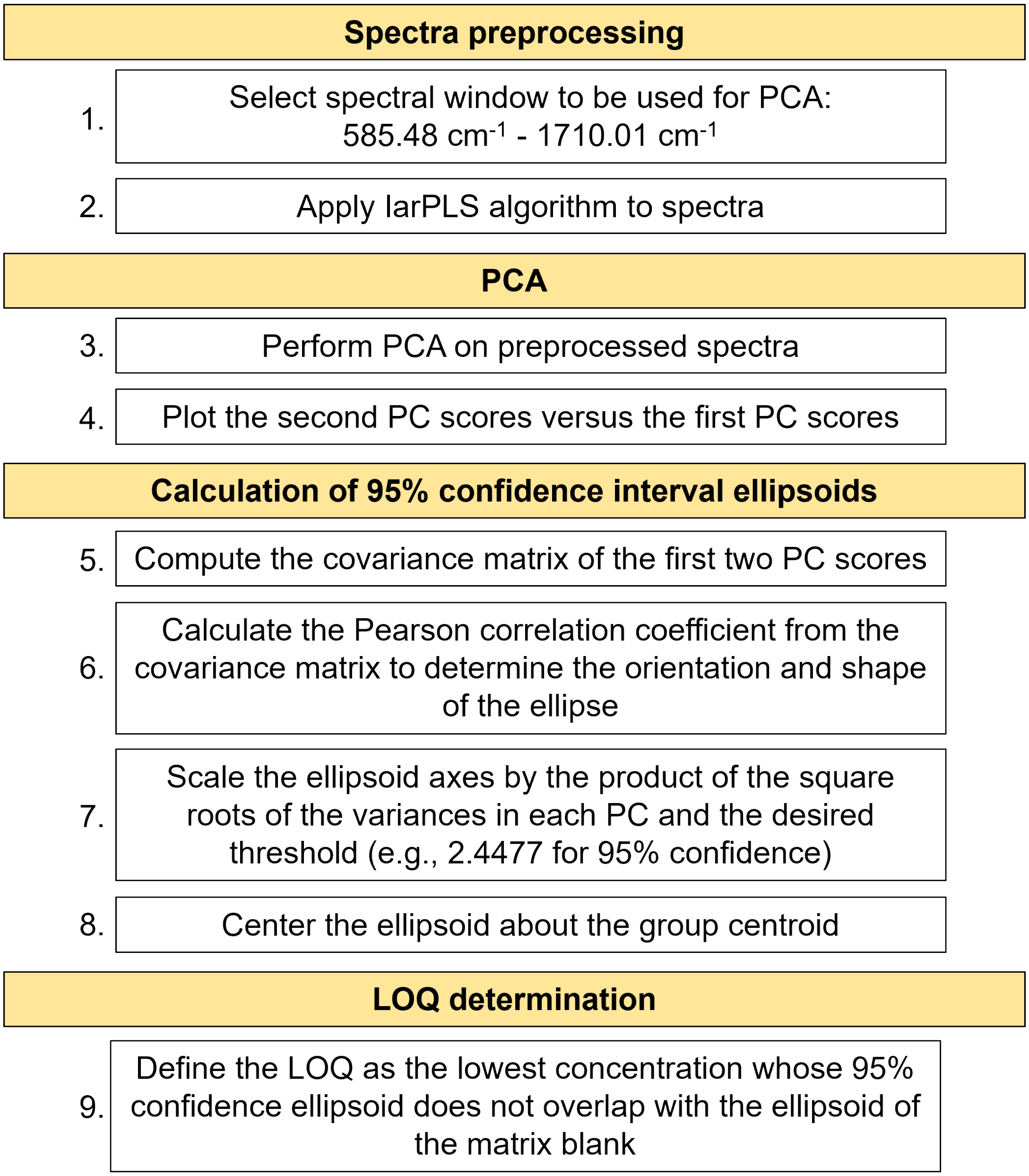

Figure S11. Overview of the PCA workflow applied in this study for LOQ determination.

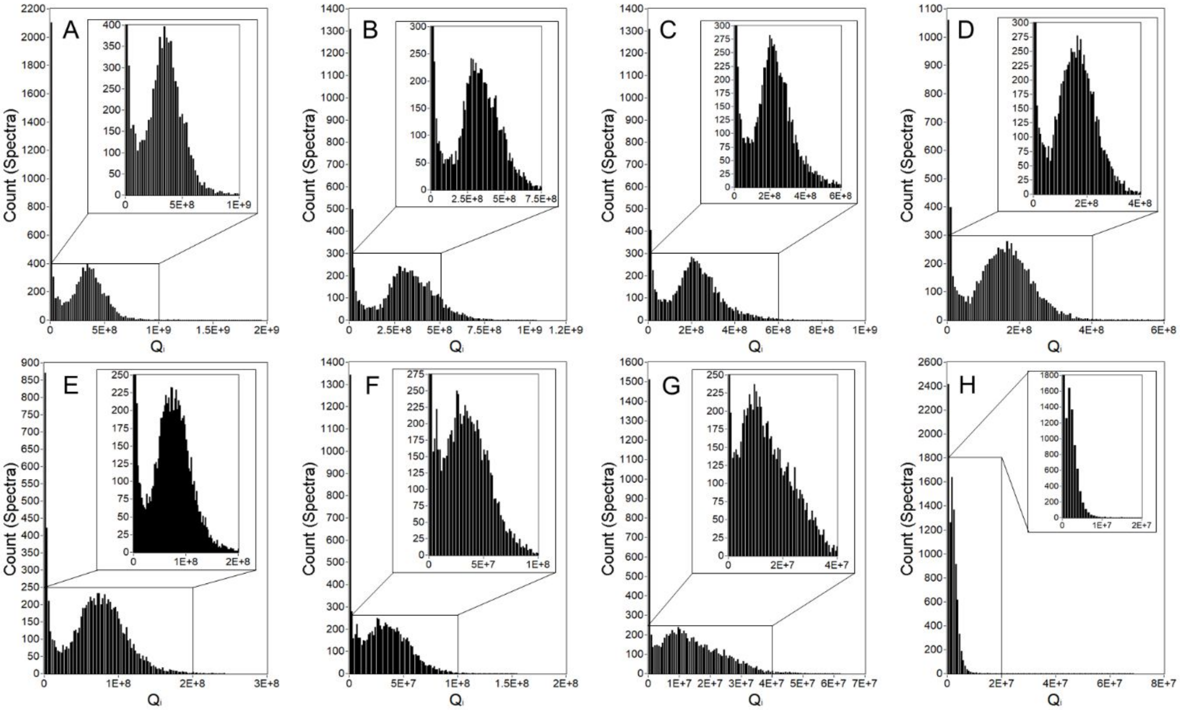

Figure S12. Histograms of the Q_i distribution of all acquired spectra for each FTC concentration in plasma of the first experiment replicate. **(A)** 5000 ng/mL; **(B)** 2500 ng/mL; **(C)** 1250 ng/mL; **(D)** 625 ng/mL; **(E)** 312 ng/mL; **(F)** 156 ng/mL; **(G)** 78 ng/mL; **(H)** Blank (non-spiked plasma). Histograms were prepared using 100 intervals where the count of spectra in each interval was plotted as a function of Q_i. The histogram bar width corresponds to the Q_i range of spectra in each interval. Zoomed insets of clustered data for each concentration are shown for clarity.

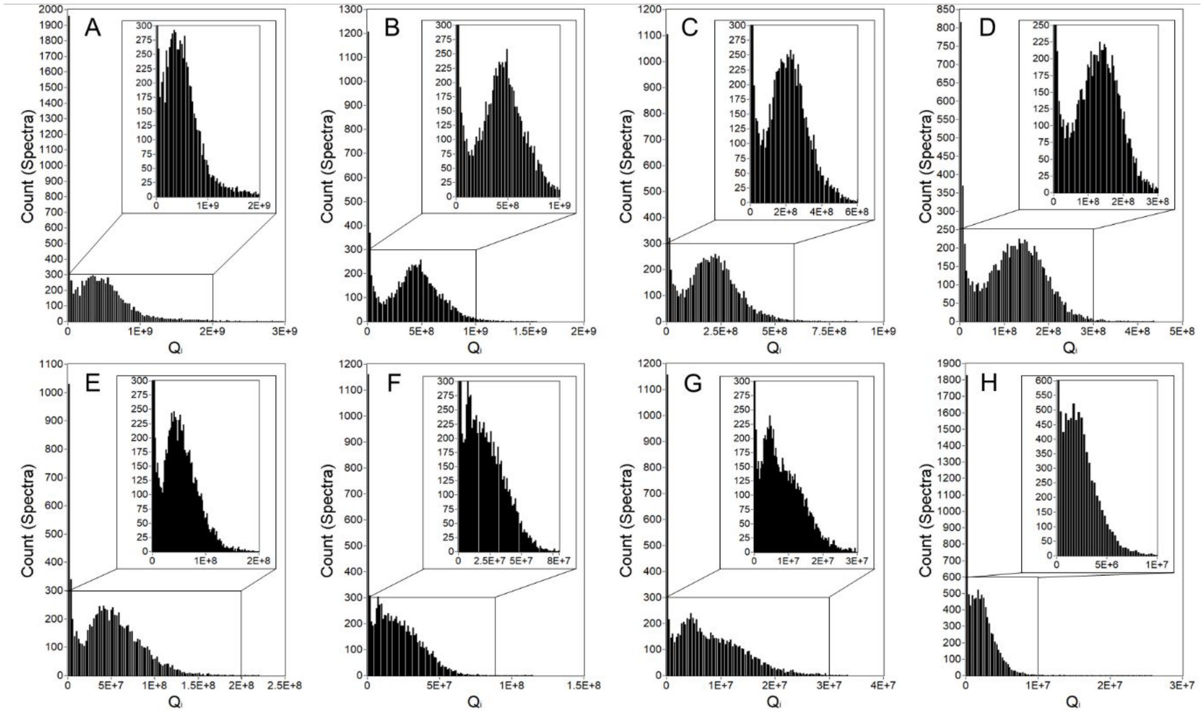

Figure S13. Histograms of the Q_i distribution of all acquired spectra for each FTC concentration in plasma of the second experiment replicate. **(A)** 5000 ng/mL; **(B)** 2500 ng/mL; **(C)** 1250 ng/mL; **(D)** 625 ng/mL; **(E)** 312 ng/mL; **(F)** 156 ng/mL; **(G)** 78 ng/mL; **(H)** Blank (non-spiked plasma). Histograms were prepared using 100 intervals where the count of spectra in each interval was plotted as a function of Q_i. The histogram bar width corresponds to the Q_i range of spectra in each interval. Zoomed insets of clustered data for each concentration are shown for clarity.

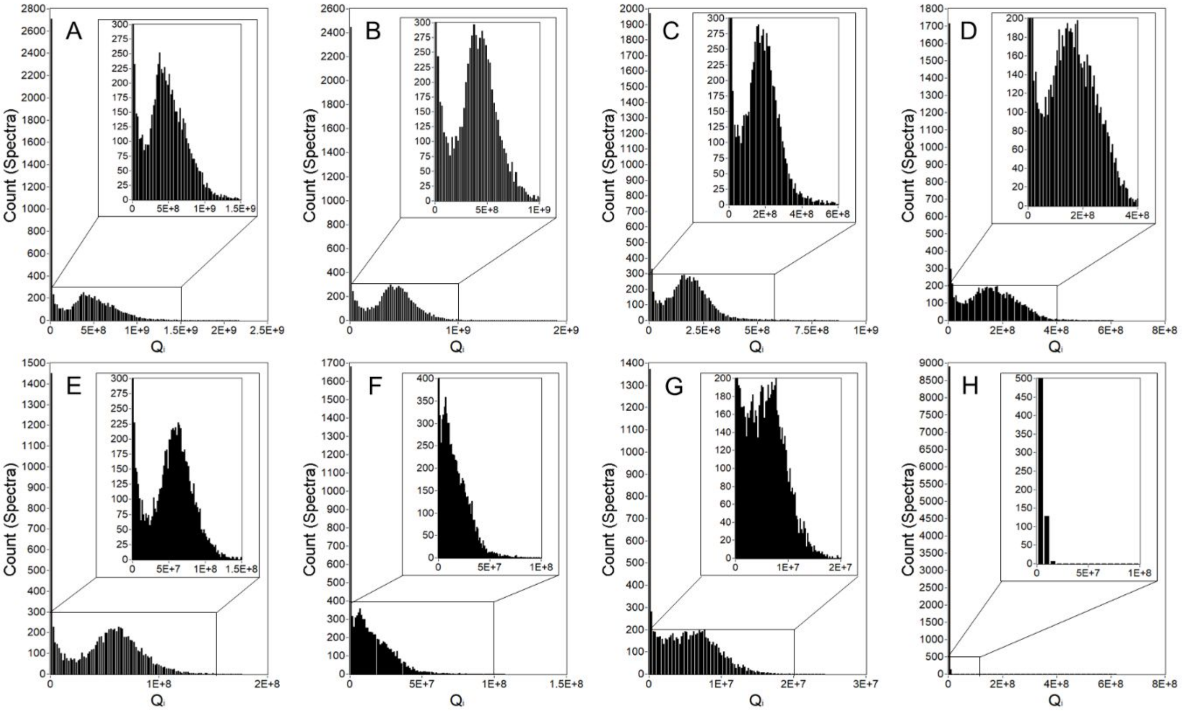

Figure S14. Histograms of the Q_i distribution of all acquired spectra for each FTC concentration in plasma of the third experiment replicate. **(A)** 5000 ng/mL; **(B)** 2500 ng/mL; **(C)** 1250 ng/mL; **(D)** 625 ng/mL; **(E)** 312 ng/mL; **(F)** 156 ng/mL; **(G)** 78 ng/mL; **(H)** Blank (non-spiked plasma). Histograms were prepared using 100 intervals where the count of spectra in each interval was plotted as a function of Q_i. The histogram bar width corresponds to the Q_i range of spectra in each interval. Zoomed insets of clustered data for each concentration are shown for clarity.

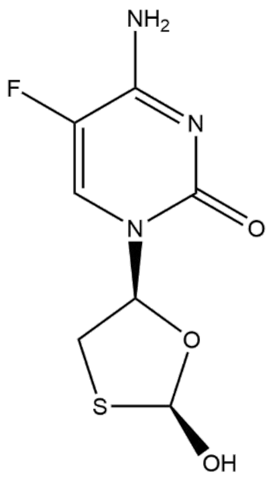

Figure S15. Chemical structure of emtricitabine (FTC).

Table S1. Peak assignments of the aqueous FTC SERS spectrum (see Fig. 6 of the main text) peaks based on literature assignments of FTC[1] and cytosine.[2]

Wavenumber of peak in aqueous FTC SERS spectrum (cm^{-1})	Literature peak (cm^{-1})	Reference	Peak assignment (see SI Fig. 11)
614	614	[1]	N – H wag
792	794	[1]	Ring breathing
968	938	[1]	C – H wag, asymmetric
1117	1119	[1]	N – H, C – H bend, asymmetric
1360	1358	[1]	N – H, C – H bend
1589	1588	[1]	NH_2, bend
1676	1676	[2]	C = C, C = N, and C = O stretch

References

(1) Hrncirova, J.; Butler, M. R.; Dutta, S.; Clark, M. R.; Cooper, J. B. Cumulative Distribution Function and Spatially Resolved Surface-Enhanced Raman Spectroscopy for the Quantitative Analysis of Emtricitabine. *Applied Spectroscopy Practica* **2024**, *2* (1). DOI: 10.1177/27551857241235972.
(2) Billinghurst, B. E.; Oladepo, S. A.; Loppnow, G. R. pH-Dependent UV Resonance Raman Spectra of Cytosine and Uracil. *The Journal of Physical Chemistry B* **2009**, *113* (20), 7392-7397. DOI: 10.1021/jp811327w.
